# A Genetically Encoded Homocysteine Precursor to Probe Protein Active Sites and to Addict *Escherichia coli* to a Noncanonical Amino Acid Directly Involved in Catalysis

**DOI:** 10.1002/anie.202509112

**Published:** 2025-07-15

**Authors:** Clara Dunker, Henning D. Mootz

**Affiliations:** ^1^ Department of Chemistry and Pharmacy Institute of Biochemistry, University of Münster Corrensstraße 36 48149 Münster Germany

**Keywords:** Catalytic residues, Enzyme mechanism, Genetic code expansion, Non‐natural amino acids, Stable disulfide

## Abstract

Noncanonical amino acids (ncAAs) incorporated into proteins by stop codon suppression are powerful tools to probe and expand protein structure and function. Although homocysteine (Hcy) is a ubiquitous, naturally occurring amino acid, it was excluded from the universal genetic code. Hcy is very interesting, yet mostly unexplored, for probing protein active sites because of its subtle structural and electronic differences from cysteine and serine, which are widespread catalytic residues in enzymes. We report the genetic encoding of a new protected Hcy precursor, HcyX, that can be conveniently deprotected by chemical reductants or bioorthogonal reagents. We find varying and sometimes remarkable levels of activity for different purified enzymes with Hcy at catalytic positions. By exploiting partial intracellular deprotection to Hcy, we show that two proteins rendered Hcy‐dependent, an intein and thymidylate synthase, can rescue growth of *Escherichia coli* by catalyzing a reaction essential for cell survival. To the best of our knowledge, these are the first examples in which cell growth is linked to a genetically incorporated ncAA directly involved in catalysis. We further demonstrate that Hcy‐based disulfide bonds are chemically more stable than cysteine disulfides. Together, these findings open new paths for the experimental evolution of the genetic code.

## Graphical abstract

A new genetically encoded amino acid is chemically converted into homocysteine (Hcy) under mild conditions to probe and to activate protein active sites. Surprisingly, Hcy can replace cysteine or serine residues in several enzymes. Its potential for a widespread use in proteins is further underlined by rendering *Escherichia coli* dependent on Hcy, providing the first link between cell survival and a noncanonical amino acid directly involved in biocatalysis.

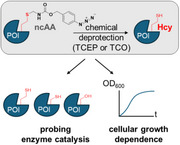

## Introduction

The universal genetic code of all known organisms evolved to encode the 20 canonical amino acids and was likely established at the time of the last universal common ancestor.^[^ ^1^ ^]^ Although the genetic code appears resistant toward changes because of the associated global detrimental effects,^[^ ^2^ ^]^ it clearly was still adaptive to evolutionary innovations, as exemplified by ambiguous codon assignments^[^ ^3^ ^]^ and the admission or loss of individual amino acids such as selenocysteine (Sec)^[^ ^4^, ^5^ ^]^ and pyrrolysine (Pyl).^[^ ^6^, ^7^ ^]^ The genetic code expansion (GCE) technology enables the genetic encoding of noncanonical amino acids (ncAAs) by co‐expressing an orthogonal pair of tRNA and aminoacyl‐tRNA synthetase (aaRS) engineered to activate the ncAA and to co‐translationally incorporate it into the protein, typically by suppressing an amber stop codon in the mRNA.^[^ ^8^, ^9^, ^10^ ^]^ NcAAs incorporated by GCE have been exploited for virtually all aspects of protein biochemistry, in particular to control and expand the structure and function of proteins in natural and unnatural ways, as well as to interrogate how their presence can alter the organismal fitness under evolutionary pressure.^[^ ^8^, ^9^, ^10^, ^11^, ^12^, ^13^, ^14^, ^15^, ^16^, ^17^, ^18^, ^19^ ^]^

We reasoned that homocysteine (Hcy) is an attractive amino acid for probing and engineering the structure and function of proteins because its reactivity, amphiphilicity, and small size^[^ ^20^ ^]^ set it apart from most other ncAAs that have been incorporated by the GCE technology.^[^ ^8^, ^21^, ^22^ ^]^ The Hcy thiol moiety is of comparable reactivity to the cysteine side chain thiol, although it is less readily deprotonated into the nucleophilic thiolate anion due to its higher p*K*_a_ of 10.0 (for the free amino acid).^[^ ^20^ ^]^ For the same reason, the Hcy thiolate is a worse leaving group than the cysteine thiolate, thereby lending more stability to disulfide bonds that involve Hcy.^[^ ^20^ ^]^ With the Cys thiol moiety being involved in many chemistries, including redox catalysis, covalent and radical reactions, metal binding, and disulfide bond formation, a wide range of similar functional roles for Hcy should be possible. The rationale for Hcy as a probe for cysteine active site residues is that its thiol group in the longer side chain is mispositioned relative to a general base or other catalytic residues. Furthermore, since the Hcy thiol group has a higher reactivity than the hydroxyl group of the serine side chain, it should also be useful to probe the specialization of protein active sites for this residue, in which case the higher reactivity might offset the subtle structural and electronic changes.

Incorporation of Hcy into proteins is also interesting because Hcy is a naturally occurring amino acid present in the metabolism of cells, like, for example, ornithine^[^ ^23^ ^]^ and citrulline.^[^ ^24^ ^]^ However, Hcy was not selected as a canonical amino acid in the early steps of life formation, probably for reasons of chemical stability, as its aminoacyl adenylate and aminoacyl‐tRNA ester are susceptible to intramolecular attack of the thiol side chain onto the activated carboxylate through a sterically favorable 5‐membered ring.^[^ ^25^ ^]^ It therefore remains largely unexplored to what extent Hcy could assume essential functions in (contemporary) enzymes.

To the best of our knowledge, there are only a couple of reports in which Cys was substituted for Hcy to probe enzyme–structure–activity relationships. Replacement of the catalytic Cys184 in the sortase A with Hcy reduced the transpeptidase activity to less than 1%.^[^ ^26^ ^]^ In this case, the noncanonical amino acid was introduced by protein semisynthesis as part of a chemically synthesized peptide that was linked to the recombinant remainder of the enzyme's polypeptide sequence by expressed protein ligation (EPL). Replacement of the Cys1 at the upstream splice junction in the split *Ssp* DnaB intein yielded approximately 7% of protein *trans*‐splicing activity.^[^ ^27^ ^]^ Again, Hcy was part of a synthetic peptide that in this case assembled noncovalently with its complementary recombinant fragment. The impairments in activity of these two proteins likely reflect not only the lower nucleophilicity of the Hcy thiol but also the evolutionary optimization of active sites to the geometry of the cysteine side chain.

**Figure 1:**
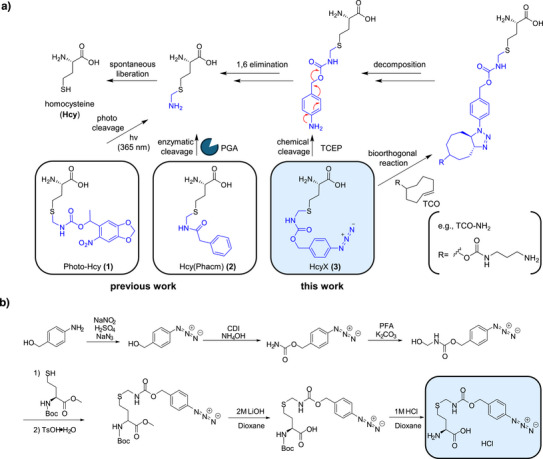
Genetically encoded homocysteine (Hcy) precursors. a) Deprotection schemes to give Hcy. HcyX (**3**) is the new ncAA described in this work that can be transformed into Hcy by the indicated reactions. b) Chemical synthesis of HcyX (**3**).

Methods for the direct genetic encoding of Hcy are missing. The development of an orthogonal aminoacyl‐tRNA synthetase specific for Hcy is challenging due to the structural similarity with Cys, Met, and other hydrophobic amino acids, as well as the mentioned thiolactone formation from the activated Hcy species.^[^ ^28^ ^]^ Both these problems can be circumvented in the genetic encoding of protected forms of Hcy that are converted to Hcy at a posttranslational stage. Deiters and coworkers designed caged Photo‐Hcy **1** (Figure 1a) that was recognized by a mutated pyrrolysyl‐tRNA synthetase.^[^ ^29^ ^]^ Following its genetic incorporation into proteins, the photolabile group is removed with UV light (365 nm), triggering a spontaneous decomposition of the remaining aminohemiacetal to the Hcy side chain. Incorporation of **1** at position Cys124 close to the active site of Renilla luciferase allowed the light‐dependent activation of this enzyme mediated through the steric relief of the removed protection group.^[^ ^29^ ^]^ To avoid the phototoxicity associated with the UV irradiation, we developed Hcy(Phacm) **2** (Figure 1a).^[^ ^30^ ^]^ The phenylacetyl moiety of the phenylacetamidomethyl (Phacm) group can be enzymatically removed with penicillin G acylase (PGA), again followed by decomposition of the aminomethyl intermediate to the free Hcy side chain. While this protocol was used to consecutively modify Cys and Hcy thiol groups as chemical handles in dual labeling approaches under mild conditions,^[^ ^30^ ^]^ it also revealed that PGA could efficiently act only on sterically well‐accessible positions in disordered peptide regions,^[^ ^30^, ^31^ ^]^ unlike those typical for more buried active site residues of enzymes. Neither **1** nor **2** were used so far for mechanistic studies and to probe catalytic Cys or Ser residues.

In this work, we report the new *para*‐azidobenzyl(oxycarbonyl)aminomethyl homocysteine (**3**), termed Hcy(PABCam) or, in short, HcyX (Figure 1a), as a genetically encoded Hcy precursor that can be chemically deprotected using reducing conditions or by a bioorthogonal reaction. By this means, we conveniently prepare proteins with Hcy even at poorly accessible positions under mild and physiological conditions without the need for damaging UV light. By substituting active site Cys and Ser residues in various enzymes, we find a surprising range of activities of the probed proteins, suggesting a significant potential of Hcy to support catalysis without further evolutionary adaptation. We further show that HcyX is deprotected in live *Escherichia coli* cells depending on reductive conditions. We explore this notion to make *E. coli* growth‐dependent on HcyX, showing to the best of our knowledge the first example of an ncAA being directly involved as key protein active site residue in biocatalysis to support cell survival.

## Results and Discussion

We designed HcyX **3** (Figure 1a) as a protected homocysteine precursor. The *p*‐azidobenzyloxycarbonyl group can be removed by converting the azide into an amine group, e.g., by reduction with a phosphine like TCEP, or bioorthogonally through an unstable triazoline cycloaddition product by using a *trans*‐cyclooctene (TCO) reagent.^[^ ^32^ ^]^ The amine group triggers 1,6‐elimination and is followed by release of CO_2_. The remaining aminomethyl intermediate is unstable and decomposes to give the free Hcy thiol side chain (Figure 1a).^[^ ^29^, ^30^ ^]^ We synthesized HcyX (**3**) according to the scheme in Figure 1b in six steps with a yield of 20% as a soluble HCl salt.

**Figure 2:**
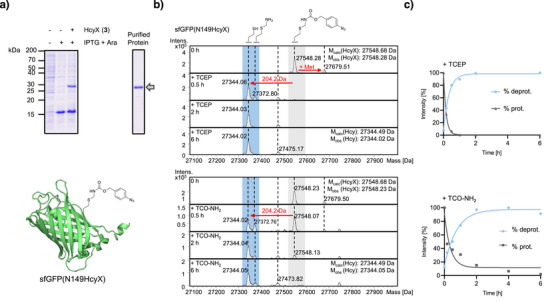
Genetic incorporation and chemical deprotection of HcyX (**3**). a) Incorporation of **3** into sfGFP at position N149. Top panels show Coomassie‐stained SDS‐PAGE gels of whole cell extracts and the purified protein, respectively. The bottom panel shows a structural illustration of sfGFP(N149HcyX) prepared using PDB‐ID 6DQ1. b) ESI‐MS analysis of chemical deprotection. Purified sfGFP(N149HcyX) was incubated with TCEP or TCO‐NH_2_ as indicated. See Figure S2 for the analysis at additional time points. c) Plots of the reactions calculated on an exemplary data set (of three repetitions) as shown in (b).

To incorporate HcyX (**3**) during translation via amber stop codon suppression, we found that a *Methanosarcina mazei* pyrrolysine tRNA synthetase (*Mm*. PylRS) with the mutations L309A, C348A, and Y384F accepted the new ncAA as a substrate. These mutations correspond to a previously reported *Methanosarcina barkeri* PylRS mutant for the incorporation of the isosteric *p*‐azidobenzyloxycarbonyl lysine (PABK).^[^ ^32^, ^33^ ^]^ We further introduced the P188G mutation to improve PylRS stability.^[^ ^34^ ^]^ We expressed sfGFP(N149HcyX)‐H_6_ (superfolder green fluorescent protein) in *E. coli* BL21 (DE3) gold cells with **3** (1 mM) added to the growth medium. Purification by Ni‐NTA chromatography afforded 2.1 mg recombinant protein from a 50 mL culture, thus exceeding previously reported expression levels using PABK^[^ ^32^, ^33^ ^]^ (Figures 2a and S1). We confirmed incorporation of HcyX (**3**) by validation of the expected mass (Figure 2b, top spectrum).

We tested deprotection of the incorporated **3** in sfGFP(N149HcyX) by either adding TCEP (1 mM) or TCO‐amine (TCO‐NH_2_; 4 mM; Figure 1a). We observed formation of sfGFP(N149Hcy) in virtually quantitative yields with the expected loss of 204.2 Da within 30 min or about 6 h, respectively (Figures 2b and S2). In both cases, the rate‐determining step was the final decomposition of the aminomethyl intermediate (Figures 1a and S2), in agreement with previous observations for Hcy(Phacm) (**2**).^[^ ^30^ ^]^ Thiol‐based reducing agents were less reactive compared to TCEP, as expected, with DTT (1 mM) showing slow deprotection overnight and GSH (5 mM) being nearly unreactive (Figure S2a).

**Figure 3:**
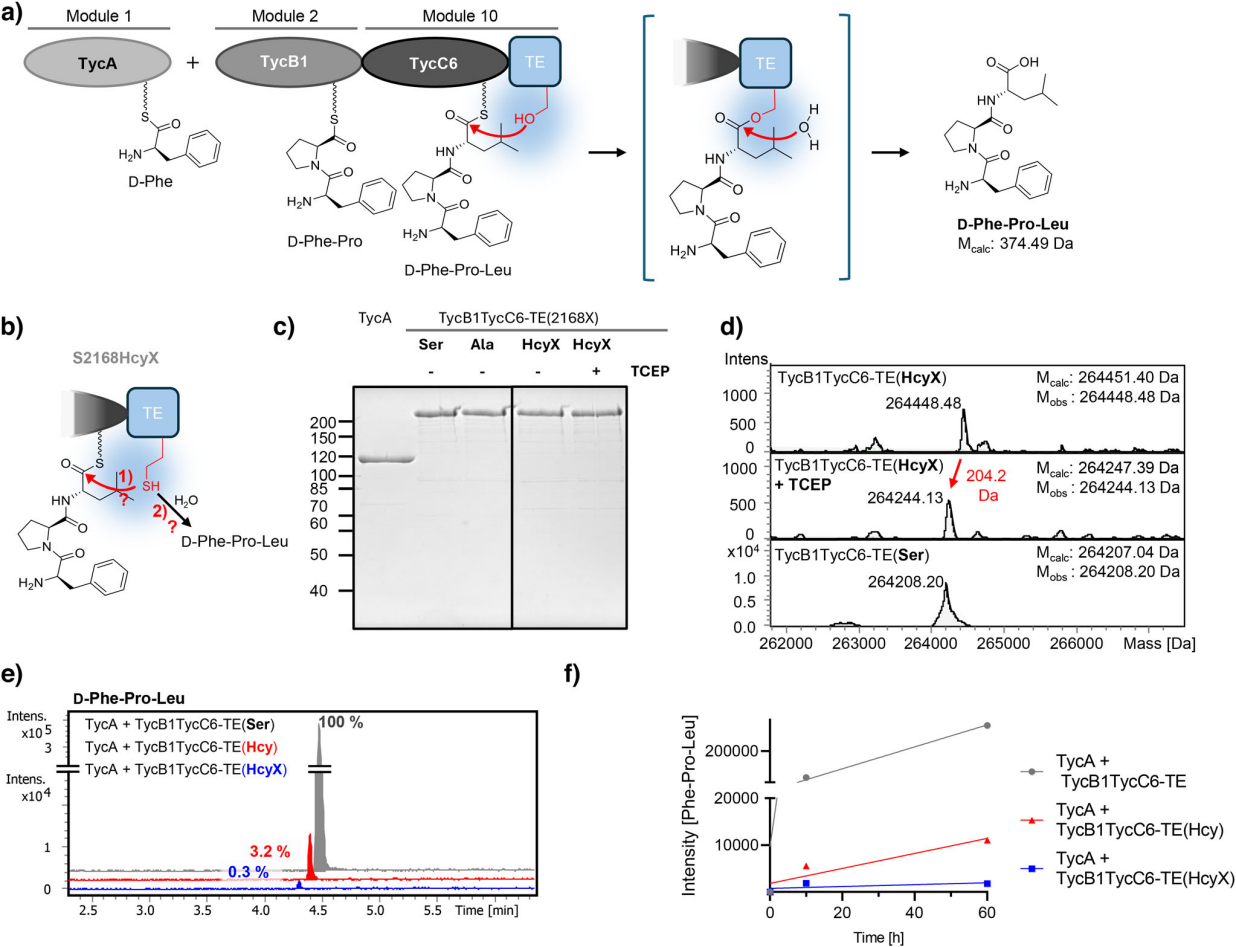
Hcy in the catalytic triad of a thioesterase (TE) domain. a) Scheme of tripeptide synthesis in a model nonribosomal peptide synthetase. b) The serine nucleophile of the TE domain was probed with Hcy as a substitute. c) Coomassie‐stained SDS‐PAGE gel of purified proteins. d) ESI‐MS analysis of the S2168HcyX mutant to confirm HcyX incorporation and deprotection. The analysis of the wildtype protein with Ser2168 is shown for comparison. e) HPLC‐MS analysis of tripeptide formation with indicated yields relative to the wildtype protein. Proteins were incubated for 180 min with ATP (5 mM), l‐Phe, l‐Pro, and l‐Leu (1 mM each). f) Time courses of the tripeptide formation reactions calculated on an exemplary data set of three repetitions.

We then set out to explore if Hcy can substitute for cysteine or serine as catalytic residue in the active sites of proteins. The TEV protease is a cysteine‐dependent peptidase that recognizes the sequence motif ENLYFQ‐(G/S).^[^ ^35^ ^]^ Asp81, His46, and Cys151 form a catalytic triad to activate Cys151 for nucleophilic attack. To probe the enzyme for activity with Hcy151 as an altered active site nucleophile, we introduced the amber stop codon in a TEV expression plasmid and incorporated **3** to give TEV(C151HcyX) (Figure S3A–C). ESI‐MS analysis confirmed the identity of both the HcyX protein as well as the successful deprotection of the Hcy side chain (Figure S3B). However, we could not detect any proteolytic activity of TEV(C151Hcy) toward a substrate protein that was readily cleaved by wildtype TEV (Figure S3D). The failure of Hcy to substitute for Cys in the framework of this enzyme indicates that the TEV active site is highly specialized to position the Cys thiol group relative to His46 as the general base and to the carbonyl carbon atom of the chemically stable peptide bond of the substrate. With this insight in mind, we thought of a Ser‐dependent hydrolase that attacks a less stable bond. We hypothesized that both the higher nucleophilicity of the Hcy side chain compared to the serine hydroxyl group and a more electrophilic carbonyl carbon at the scissile bond might shift the activity profile in favor of the Hcy‐containing protein. The thioesterase (TE) domain of nonribosomal peptide synthetases (NRPS) employs a catalytic triad (Asp‐His‐Ser) to attack a peptidyl‐thioester substrate assembled by the multidomain enzymes on the 4′‐phospho‐pantetheinyl prosthetic group (Ppant) of the last module.^[^ ^36^ ^]^ The formed peptidyl‐*O*‐acyl intermediate is then either hydrolyzed through water as a nucleophile or cyclized through a functional group in the peptidyl moiety as a nucleophile.^[^ ^37^ ^]^ We investigated a shortened model system of the tyrocidine NRPS in which modules three to nine were deleted (Figure 3a).^[^ ^38^ ^]^ The last module, 10, carries the TE domain that hydrolyzes the tripeptide d‐Phe‐l‐Pro‐l‐Leu synthesized by the modules 1, 2, and 10 (TycA + TycB1TycC6‐TE). We mutated the catalytic Ser2168 in the TE domain to Ala and to an amber stop codon to incorporate HcyX (Figures 3b–d and S4A). We confirmed by ESI‐MS that treatment of the 264 kDa TycB1TycC6‐TE(HcyX) protein with TCEP resulted in HcyX deprotection to give TycB1TycC6‐TE(Hcy) with the characteristic mass difference of 204.2 Da (Figures 3d and S4A). We then analyzed the catalytic activities of the mutant proteins by HPLC‐MS analysis of the formed tripeptides following incubation with ATP and the substrate amino acids l‐Phe, l‐Pro, and l‐Leu. With the deprotected TycB1TycC6‐TE(Hcy), we found a low, yet remarkable tripeptide formation relative to the wildtype enzyme with the Ser nucleophile (3.2% after 60 min; Figure 3e,f). In contrast, the protected enzyme TycB1TycC6‐TE(HcyX) formed only background levels (0.3%) of tripeptide (Figure 3e,f), similar to a S2168A control mutant (0.7%) (Figure S4B), as expected. These residual activities of the HcyX and Ala mutants are likely due to some noncatalyzed cleavage of the peptidyl‐Ppant thioester. Thus, Hcy was clearly capable of participating as active site nucleophile in the covalent catalysis of this catalytic triad, despite its longer side chain compared to the native serine.

**Figure 4:**
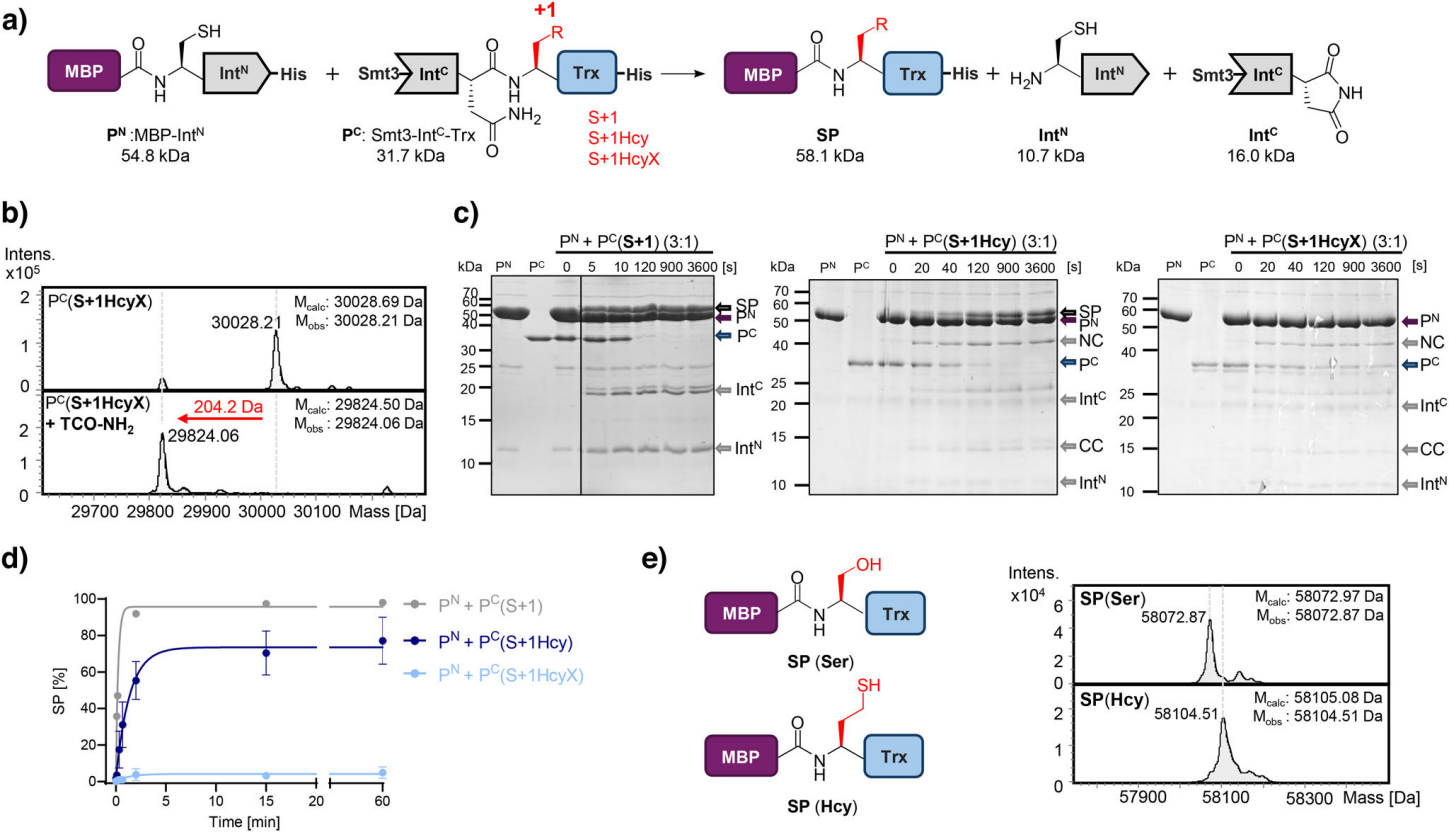
Hcy‐dependent protein *trans*‐splicing. a) Scheme of the protein *trans*‐splicing assay. b) ESI‐MS of the Smt3‐Int^C^(S+1HcyX)‐Trx‐H_6_ C‐terminal precursor (P^C^) and its chemical deprotection of the HcyX side chain. c) Protein *trans*‐splicing assays of P^N^ and P^C^ precursors in a 3:1 ratio, using wildtype (S+1) and mutant (S+1Hcy and S+1 HcyX) P^N^ precursors as indicated. Shown are exemplary Coomassie‐stained SDS‐PAGE gels. d) Time courses of the reactions determined by densitometric analysis of protein bands as shown in (c). Error bars represent the standard deviation of three technical repeats. e) ESI‐MS spectra of obtained S+1 and S+1Hcy splice products (SP). NC = N‐cleavage product (MBP); CC = C‐cleavage product (Trx‐His).

We next asked whether Hcy could participate as an active site residue at the C‐terminal splice junction in protein splicing. Inteins are single‐turnover enzymes that catalyze their own removal out of a precursor protein and this reaction involves covalent catalysis through thioester or oxyester intermediates at their 1 and +1 positions (Figure 4a).^[^ ^39^, ^40^ ^]^ Split inteins are easier to investigate because their protein *trans*‐splicing reaction can be started by mixing the N‐ and C‐terminal precursors (P^N^ and P^C^). Figure S5 shows all steps of the splicing mechanism of the benchmark split Gp41‐1 intein,^[^ ^41^ ^]^ which operates with Cys1 and Ser+1 residues at the splice junctions. The Ser+1 residue attacks the initially formed Cys1 thioester at the N‐terminal splice junction to give a branched oxyester intermediate. The acyl group on the Ser+1 side chain then shifts to the Ser+1 α‐amino group once the latter has been liberated by the cleavage of the scissile peptide bond at the downstream splice junction. Notably, the importance of a general base or another catalytic mechanism to promote the Ser+1 hydroxyl group attack on the thioester, apart from positioning the interacting groups, is still under debate.^[^ ^39^, ^42^, ^43^ ^]^ A mutant protein with a S+1C substitution to the more reactive cysteine still supports splicing.^[^ ^44^ ^]^ We therefore thought to probe the intein active site tolerance toward the subtle structural changes of a Ser+1 substitution with Hcy. We prepared Smt3‐Int^C^(S+1HcyX)‐Trx as the C‐terminal precursor P^C^ of the split Gp41‐1 intein. Thioredoxin (Trx) served as a model extein and yeast Smt3 as an expression tag. Following treatment with TCEP, we confirmed by ESI‐MS the efficient deprotection of HcyX to Hcy to give Smt3‐Int^C^(S+1Hcy)‐Trx (Figure 4b). Surprisingly, we observed protein *trans*‐splicing with very high efficiency. When incubated with MBP‐Int^N^ as the complementary P^N^, the expected splice product MBP‐Hcy‐Trx‐His_6_ (SP) was formed at about 77% yield with a rate of the pseudo‐first‐order reaction of only about five‐fold lower than that of the wildtype P^C^ (*t*_1/2_ = 42.5 s and *t*_1/2_ = 9 s,^[^ ^41^, ^45^ ^]^ respectively; Figure 4c,d). The loss in splice product yield compared to the wildtype (90%–95%, Figure 4c,d) can be largely explained by the increased formation of the C‐cleavage product (CC) at about 18%, which is likely a consequence of the slight misalignment in the active site due to the structural and electronic changes of the S+1Hcy substitution. A control reaction showed that the protected S+1HcyX precursor was inactive in protein *trans*‐splicing, as expected (Figure 4c,d). Our scheme thus also represents a new way to chemically trigger the activity of a split intein through caging of one of the nucleophilic side chains involved in catalysis.^[^ ^46^, ^47^ ^]^

**Figure 5:**
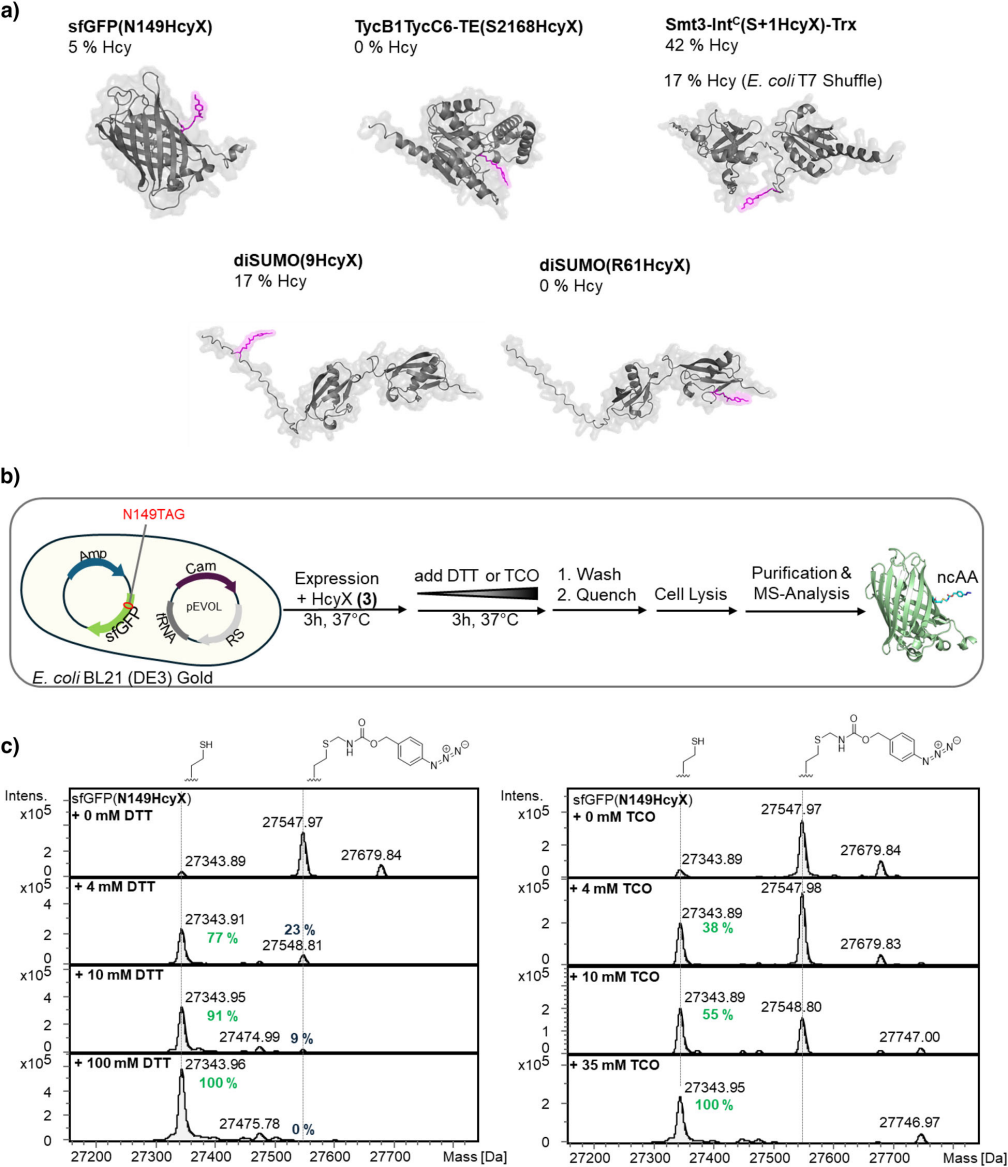
Deprotection of HcyX to Hcy *in cellulo*. a) Shown are yields of deprotected forms of proteins with Hcy at the indicated position following expression in either *E. coli* BL21 (DE3) or T7 SHuffle (DE3) cells. b) Schematic setup for assaying enforced chemical deprotection *in cellulo* in *E. coli* BL21 (DE3). c) ESI‐MS analysis of deprotection of sfGFP (N149HcyX) as shown in (b).

Interestingly, in our initial attempts to purify the above‐mentioned Smt3‐Int^C^(S+1HcyX)‐Trx construct, we observed that the HcyX side chain was partially converted to the free thiol group of Hcy (42%) when the protein was expressed in *E. coli* BL21 (DE3) cells (Figure S6A). We concluded that *in cellulo* reduction of the azide into an amine group could have triggered the premature removal of the protection group. To test this hypothesis, we performed the expression in *E. coli* T7 SHuffle (DE3) cells, which exhibit a less reductive cytoplasmic milieu.^[^ ^48^, ^49^ ^]^ Indeed, Smt3‐Int^C^(S+1HcyX)‐Trx expressed in this host was found to remain in protected form at significantly higher levels (only 17% deprotected) (Figures 5 and S6A). We tested HcyX incorporation in various other proteins using both BL21 (DE3) and T7 SHuffle (DE3) cells and observed that partial deprotection to Hcy in BL21 (DE3) cells correlated with steric accessibility, which is high in case of the S+1 position of small and largely unstructured C‐terminal intein precursor. In contrast, the N149 position on the flat surface of the rigidly folded sfGFP provides lower accessibility and yields a stable HcyX side chain (Figures 2 and 5a). Similar dependencies were observed for HcyX in two different positions of a SUMO2 dimer, either in the flexible N‐terminal tail (position 9; Hcy at 17% in BL21 cells) or on the surface of the stably folded globular second SUMO2 domain (position R61; Hcy at 0% in BL21 cells) (Figures 5a and S6B). Also, for the above‐described HcyX residues in the TEV protease and TE domain constructs (Figure S3B), no premature *in cellulo* deprotection to Hcy was observed, consistent with their low accessibility in the active site. Notably, *E. coli* T7 SHuffle (DE3) cells provide access to proteins with more stable HcyX side chains.

We next attempted to chemically deprotect HcyX in live *E. coli* cells. To this end, we added either DTT or TCO‐NH_2_ for 3 h at various concentrations to *E. coli* BL21 (DE3) cells expressing sfGFP(N149HcyX), washed the cells and lysed them in presence of excess concentrations of 4‐azidoaniline as a quencher (Figure 5b). Indeed, the purified proteins showed a concentration‐dependent deprotection of HcyX, which reached completion with 100 mM of DTT and 35 mM of TCO, respectively (Figure 5c).

Inspired by the findings of partial *in cellulo* HcyX side chain deprotection, we asked whether Hcy‐mediated enzyme catalysis could also be observed in live cells and then ultimately be used to addict cells to the ncAA HcyX (**3**) for growth. Indeed, following expression in BL21 (DE3) cells, without the addition of DTT or TCO‐NH_2_, of a fused variant of the Gp41‐1 intein with the S+1HcyX substitution in the construct MBP‐intein (S+1HcyX)‐Trx‐H_6_, we could confirm the presence of the splice product MBP‐Hcy‐Trx‐H_6_ (Figure S7). These results were consistent with at least partial HcyX deprotection *in cellulo*, which subsequently triggered protein splicing.

**Figure 6:**
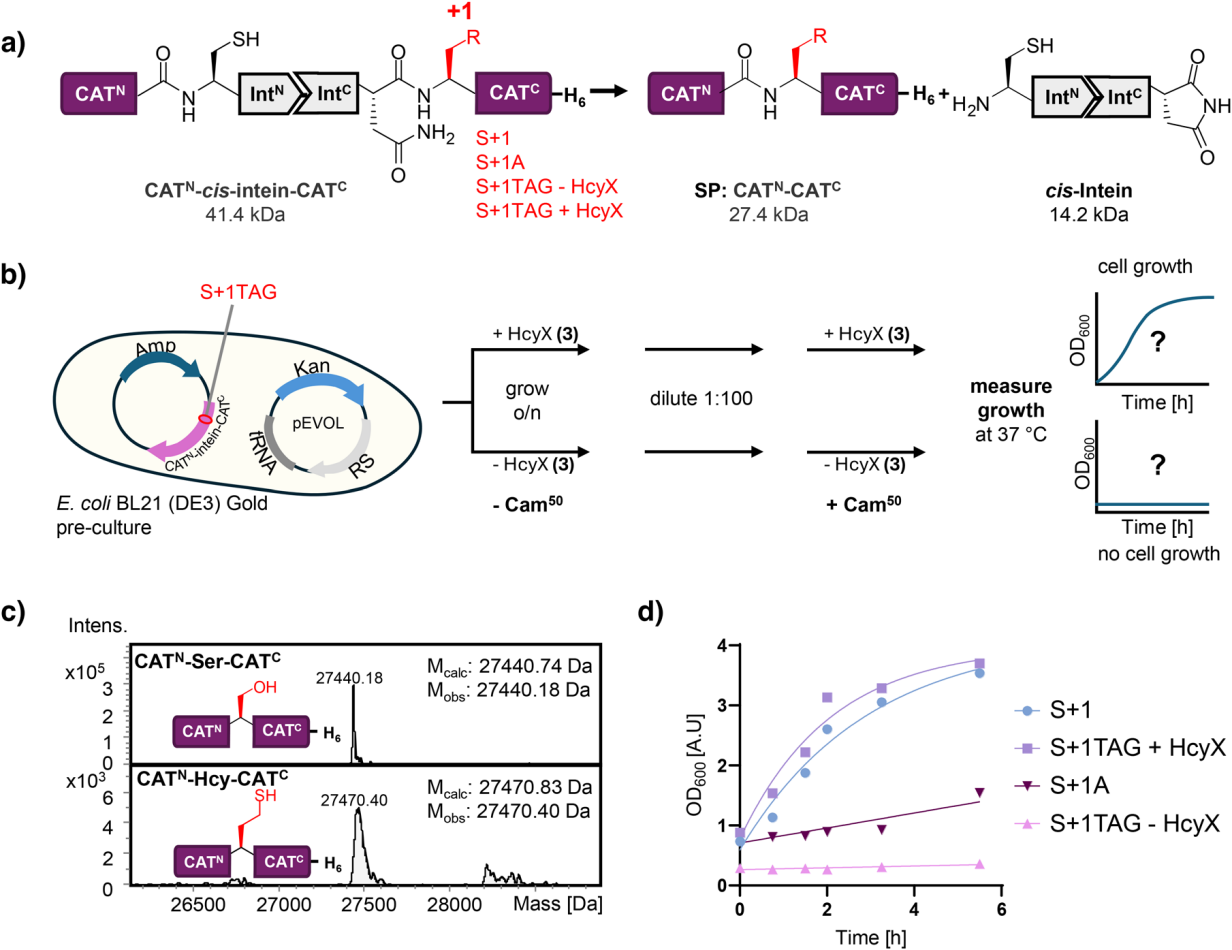
Hcy‐mediated catalysis to rescue cell growth under antibiotic pressure. a) Scheme of Hcy‐dependent protein splicing to reconstitute active CAT. b) Workflow to accumulate CAT^N^‐Hcy‐CAT^C^ before exposing cells to the selective pressure of the antibiotic Cam. c) ESI‐MS analyses of Hcy‐dependent CAT splice product (SP) and its Ser‐wildtype control. d) Growth curves of *E. coli* BL21 (DE3) cells in presence of Cam (50 µg µL^−1^). Shown are curves from an exemplary data set that was repeated three times.

With the aim to addict the cells to the Hcy‐dependent intein activity, we then genetically inserted the intein (S+1HcyX) into chloramphenicol acetyltransferase (CAT), such that intein‐mediated splicing is required to produce the intact and active antibiotic resistance protein (Figure 6a).^[^ ^50^ ^]^ Thereby, (partial) posttranslational intracellular deprotection of HcyX should render the *E. coli* BL21 (DE3) cells resistant against chloramphenicol (Cam), whereas cells not incorporating HcyX into the intein active site or not sufficiently converting it to Hcy should be unable to grow in presence of Cam (Figure 6b). Unfortunately, the expression of the CAT^N^‐intein(S+1HcyX)‐CAT^C^ construct was very weak and barely detectable. Nevertheless, we were able to confirm by ESI‐MS the formation of the spliced CAT^N^‐Hcy‐CAT^C^ with its expected increase by 30 Da compared to splice product CAT^N^‐Ser‐CAT^C^ from the wildtype control experiment (Figures 6c and S8C). Most importantly, the cells forming CAT^N^‐Hcy‐CAT^C^ grew under selective conditions in presence of Cam: Following a preincubation period of the strains carrying the amber stop codon plasmid with or without HcyX (**3**) in the absence of Cam, to allow for CAT^N^‐Hcy‐CAT^C^ to accumulate, we diluted cells in fresh growth medium with Cam (50 µg µL^−1^) added (Figure 6b). We then observed cell growth for those cells to which **3** was added, similar to the wildtype S+1 control strain, whereas cells without **3** did not show growth, comparable to the S+1A negative control strain (Figure 6d). The same experiment using T7 SHuffle cells led to no cell HcyX‐dependent growth in presence of Cam, consistent with the lower capability of this strain to post‐translationally convert the side chain into the free Hcy thiol (Figure S8A,B). Together, Hcy‐mediated catalysis rescued cell growth under selective conditions.

**Figure 7:**
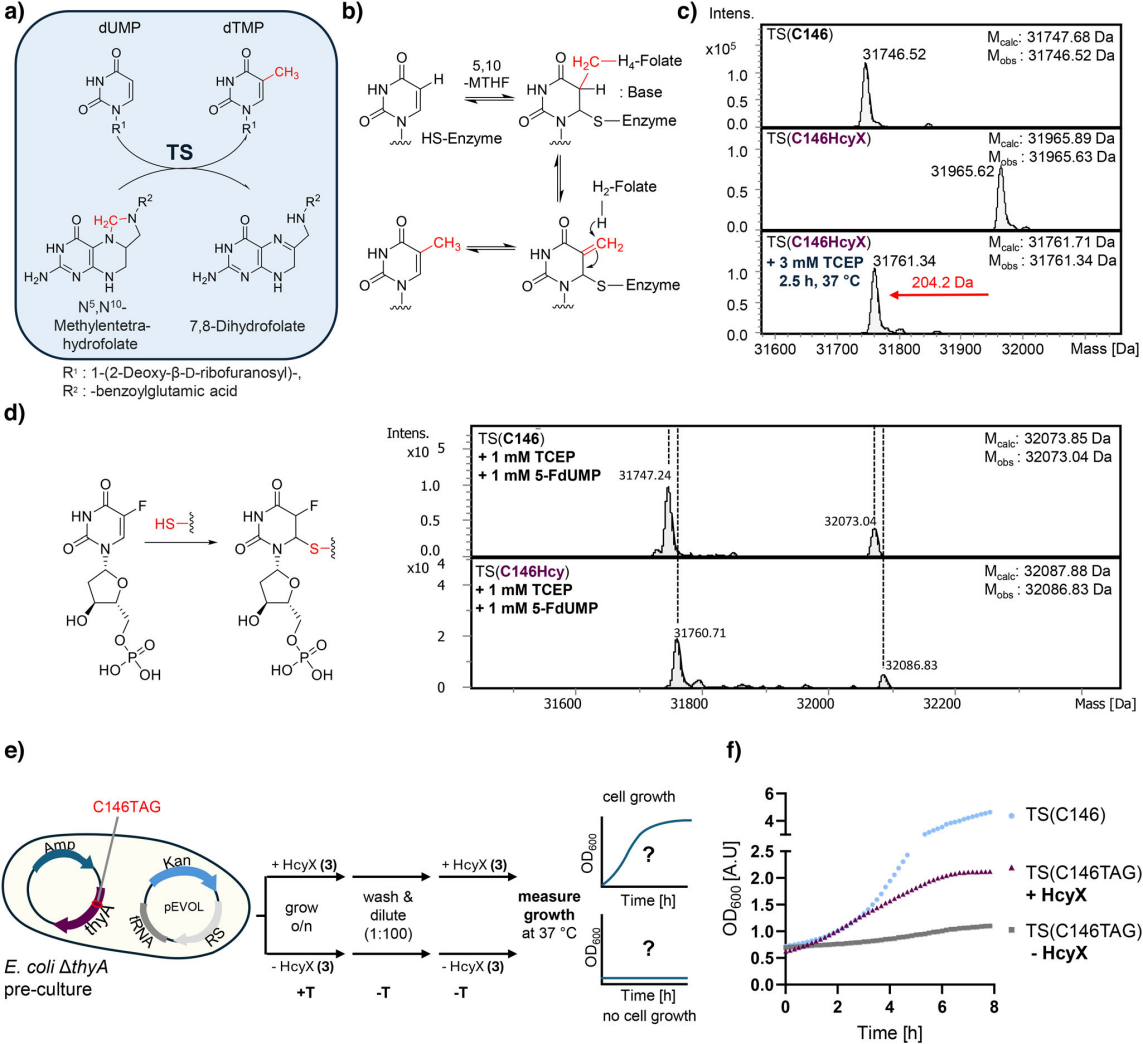
Hcy as active site residue in thymidylate synthase (TS). a) Reaction catalyzed by TS. b) Reaction mechanism of TS involving covalent catalysis of Cys146. c) ESI‐MS analysis of purified TS(C146), TS(C146HcyX), and chemical deprotection of the latter. d) Mechanism of inhibitor 5F‐dUMP (left panel) and ESI‐MS analyses of its reaction with TS(C146) and TS(C146Hcy) (right panel). e) Experimental workflow for Hcy‐dependent rescue in *E. coli* Δ*thyA* growth assay. f) Growth curves obtained from assay as described in (e). Shown are curves from an exemplary data set that was repeated three times.

Furthermore, to link *E. coli* growth to Hcy‐mediated catalysis by one of its own housekeeping proteins, we explored the key active site residue of thymidylate synthase (TS), encoded by the *thyA* gene. TS catalyzes the reductive methylation of dUMP to dTMP with 5,10‐methylenetetrahydrofolate (5,10‐MTHF) serving as a one‐carbon donor and reductant (Figure 7a).^[^ ^51^ ^]^ The essential Cys146 initiates the catalytic process by attacking the 6‐position of dUMP as a nucleophile (Figure 7b).^[^ ^52^, ^53^ ^]^ Because TS is an essential enzyme for DNA precursor biosynthesis, a *thyA* knockout strain either requires supplementation of thymine precursors like T or dT or a functional complementing gene.^[^ ^54^ ^]^ We thus asked whether a TS(C146Hcy) mutant could support cell growth in *E. coli* thymidine auxotrophy strain β1308 (Δ*thyA::erm^+^*)^[^ ^54^ ^]^ in the absence of a supplemented thymidine precursor. We first expressed and purified TS(C146HcyX) and showed that it could be quantitatively converted into the deprotected form TS(C146Hcy) using TCEP (Figures 7c and S9A,B). Figure 7d shows that both wildtype TS(C146) and TS(C146Hcy) mutant formed a covalent adduct when incubated with the suicide inhibitor 5‐F‐dUMP, indicating the Hcy side chain could attack the uracil base despite its slight mispositioning relative to its cysteine counterpart. We obtained further support for the biochemical activity of TS(C146Hcy) by showing its capability to convert uridine into thymidine in a 5,10‐MTHF‐dependent manner, similar to the wildtype TS(C146), whereas the protected TS(C146HcyX) mutant was inactive in this transformation (Figure S10). TS(C146Hcy) thus represents another example of a direct participation of Hcy's side chain in enzyme catalysis.

For cellular growth assays, we then prepared a thymine‐supplemented pre‐culture of *E. coli* β1308 (Δ*thyA::erm^+^*) harboring expression plasmids encoding both the TS(C146TAG) allele and the orthogonal tRNA/ncAARS pair for HcyX incorporation. The culture was then divided and grown overnight in the presence of thymine, either with or without HcyX (**3**) (1 mM). During this time, without selective pressure, the cells were allowed to accumulate partially deprotected TS(C146Hcy), as confirmed by MS analysis (Figure S11). Following washing to remove excess thymine, cells from each culture were then diluted 100‐fold to inoculate thymine‐less cultures, again with or without supplemented HcyX (**3**) (1 mM), and cell growth was monitored over time during further incubation at 37 °C (Figure 7e). Indeed, we observed HcyX‐dependent cell growth, albeit to a lesser degree than for the wildtype control (cells expressing TS(C146)) (Figure 7f). This result confirmed that at least partial *in cellulo* deprotection to TS(C146Hcy) had occurred and showed that a Hcy‐dependent covalent catalysis in the framework of the TS active site rescued the thymidine auxotrophy phenotype in live *E. coli* cells.

Finally, we aimed to experimentally demonstrate the formation of more stable disulfide bonds as another useful application of our new method to install Hcy residues into recombinant proteins. Following the TCO‐ or TCEP‐mediated deprotection of sfGFP(N149HcyX) we introduced disulfide bonds with either free Hcy or Cys using 2,2′‐dithiobis(5‐nitropyridine) (DTNP) to give the proteins sfGFP_Hcy‐Hcy and sfGFP_Hcy‐Cys. For comparison with the cysteine‐based disulfide, we produced the sfGFP(N149C) mutant and prepared sfGFP_Cys‐Hcy and sfGFP_Cys‐Cys. Figure S12 shows that indeed the Hcy‐based disulfide bond in sfGFP_Hcy‐Hcy was significantly more resistant toward reduction with DTT than its cysteine‐based counterpart in sfGFP_Cys‐Hcy (stability decreasing in the order Hcy‐Hcy ≥ Hcy‐Cys >> Cys‐Hcy ≥ Cys‐Cys).

## Conclusion

We have described a new approach to synthesize recombinantly produced proteins with the naturally occurring, yet noncanonical amino acid Hcy. Hcy was genetically incorporated by the GCE technology in the protected form of HcyX (**3**) to circumvent the difficulties associated with its direct recognition by an aminoacyl‐tRNA synthetase and with the chemical instability of activated Hcy‐AMP and Hcy‐tRNA intermediates. Importantly, HcyX can be chemically deprotected to Hcy under mild conditions using reductive or bioorthogonal chemistry, which has the advantages of avoiding potential photo damage inferred from the previously reported ncAA Photo‐Hcy (**1**), which requires UV irradiation to remove its photolabile protection group, and of overcoming limitations when removing the enzymatically labile protection group of previously reported ncAA Hcy(Phacm) (**2**) at sterically hindered positions.

Using HcyX incorporation and its chemical deprotection, we introduced Hcy‐based disulfides into proteins, to the best of our knowledge for the first time. We demonstrated their increased stability against reducing agents when compared to cysteine disulfides, which will, together with their extended length,^[^ ^55^ ^]^ open up new protein design opportunities.

As the main part of this work, however, we prepared several purified proteins with Hcy substituting catalytic Cys or Ser residues to probe to what degree the active site can tolerate the subtle electronic and structural alterations. The finding that all Hcy proteins were less active than their wildtype counterparts is not surprising given their expected evolutionary optimization for the native Cys or Ser residues. Rather, it is remarkable how much Hcy‐mediated activity we observed when replacing the active site Ser in the thioesterase domain of NRPSs (ca. 3% compared to wildtype) or when replacing the Ser+1 at the downstream splice junction of a split intein (ca. 77% efficiency at a five‐fold lower rate compared to the wildtype). To the best of our knowledge, these are the highest activities of Hcy‐dependent protein catalysts reported so far.^[^ ^26^, ^27^ ^]^ Also, the substitution of the active site Cys of thymidylate synthase to Hcy yielded a functional enzyme capable of reacting with the covalent suicide inhibitor 5‐F‐dUMP, however, we did not quantify its activity relative to the wildtype. Only the substitution of the active site Cys of TEV protease for Hcy resulted in an obviously inactive protein catalyst, indicating the requirement for a highly specialized alignment of residues in this active site. Notably, in all these investigated enzymes the probed residues are involved in covalent catalysis with their substrates. The ncAA Hcy thus directly participates as an essential residue in catalysis. Together, these findings suggest that Hcy‐dependent enzyme catalysis can be possible in many contemporary protein scaffolds, even without adapting them to the ncAA by directed evolution or molecular design. Our results also show that HcyX (**3**) incorporation can be utilized to create cysteine‐ or serine‐dependent proteins in an inactive pro‐form, which can then be chemically activated.

We further demonstrated that Hcy‐dependent proteins could even be linked to cell survival, such that bacterial growth relied on the catalysis mediated by the ncAA. These experiments were made possible by the partial spontaneous cellular deprotection of HcyX (**3**) to Hcy, likely by the reductive milieu in the cytoplasm that can convert the azide moiety into an amino group. We observed rescue of *E. coli* growth under selective conditions based on the splicing activity of the Hcy‐dependent Gp41‐1 intein inserted in the CAT protein for chloramphenicol resistance as well as on the Cys146Hcy mutant of thymidylate synthase as an essential enzyme in DNA metabolism. To increase the intracellular yields of Hcy‐proteins for such in vivo approaches and to support infinite growth, the discontinuous scheme of HcyX incorporation and posttranslational deprotection will require further improvement in the future. Notably, also the free ncAA HcyX can be deprotected and thereby will be lost for recognition and charging onto the respective tRNA. A similar intracellular deprotection was observed for the *p*‐nitrocarbobenzyloxy group, for which the elimination is thought to be triggered by endogenous nitroreductases.^[^ ^56^, ^57^ ^]^

Linking cell growth to the incorporation and catalytic participation of an ncAA is a challenging task that, to the best of our knowledge, has never been demonstrated so far as directly as in this work. While ncAAs are often used to probe catalytic mechanisms of enzymes,^[^ ^8^, ^18^, ^19^ ^]^ attempts to do so with proteins essential for cell growth in an in vivo setting are very rare. Despite many studies that reported the construction of *E. coli* strains that depend on genetically encoded incorporation of a particular ncAA, in most of these reports, the ncAA is not associated with catalysis important for cell survival but rather only serves to ensure translation of the full‐length version of an essential protein or its proper folding. An example of the importance of the simple suppression of the stop codon is the selection of an ncAARS with new substrate specificity by incorporation of the ncAA, irrespective of its side chain, into a permissive position of a marker protein like CAT.^[^ ^58^ ^]^ Making the folding of an essential protein dependent on an ncAA can create a cell‐dependence on a specific ncAA side chain, in which case the sequence of the protein is remodeled around the incorporated ncAA by directed evolution. For example, to create synthetic auxotrophy for biocontainment, protein mutants were selected to be dependent on an ncAA of unusual size and structure at a given position for the correct folding of the protein.^[^ ^59^, ^60^, ^61^, ^62^, ^63^ ^]^ Another more elaborate design relies on intramolecular crosslinking of the ncAA side chain that renders the essential protein sufficiently thermostable under selective conditions.^[^ ^64^, ^65^ ^]^ However, in none of these cases the ncAA is directly participating as a catalytic residue. We found only a few reports in which the ncAA was more directly part of the active site of the essential enzyme. Schultz and coworkers incorporated acetyl lysine at the position of a critical lysine in an essential branched‐chain aminotransferase. Cell growth was dependent on the ncAA incorporation and its sirtuin‐catalyzed deacetylation to lysine. Thus, the actual catalysis was not performed by an unnatural amino acid.^[^ ^66^ ^]^ The Schultz group also described a remodeled mannose‐6‐phosphate isomerase that relied on methyl‐histidine to coordinate the Zn^2+^ ion essential for catalysis,^[^ ^67^ ^]^ thus this ncAA was at least important for catalysis in the second shell of the enzyme active site. Marlière and coworkers showed that the positively charged azaleucine can rescue the loss of an arginine side chain that coordinates the substrates in thymidylate synthase.^[^ ^68^ ^]^ However, azaleucine was not genetically encoded in a specific way but incorporated using the unspecific selective‐pressure method.

On the other hand, several designer enzymes were reported that rely on the direct catalytic participation of an ncAA with unique chemical properties as active site residue.^[^ ^18^, ^19^, ^69^, ^70^, ^71^, ^72^, ^73^ ^]^ However, the reactions catalyzed by these designer enzymes are more interesting as model transformations and have not been linked to cell growth so far.

Taken together, we show that Hcy can functionally replace catalytically active Cys and Ser residues in protein active sites and hence is a useful tool to mechanistically probe them. We demonstrate its direct link to cell survival, i.e., the possibility to addict *E. coli* to Hcy based on its catalytic properties when genetically encoded in proteins. Such an experimental setup may help to further evolve the genetic code^[^ ^17^, ^74^ ^]^ in a simulation of a scenario when other amino acids were selected based on their functional merits in proteins, i.e., selenocysteine and pyrrolysine.

## Supporting Information

The authors have cited additional references within the Supporting Information.^[^ ^75^, ^76^, ^77^, ^78^, ^79^, ^80^, ^81^ ^]^

## Conflict of Interests

The authors declare no conflict of interest.

## Supporting information



Supporting Information

## Data Availability

The data that support the findings of this study are available from the corresponding author upon reasonable request.
