# Supplementary material for: A Genetically Encoded Homocysteine Precursor to Probe Protein Active Sites and to Addict Escherichia coli to a Noncanonical Amino Acid Directly Involved in Catalysis
Source: Angew Chem Int Ed Engl. 2025 Jul 15;64(36):e202509112. doi: 10.1002/anie.202509112 (PMC12402908; doi:10.1002/anie.202509112)
Supplement: Supplementary file 1 — Supporting Information [file ANIE-64-e202509112-s001.pdf]

Table of Contents

Supporting Figures………………………………………………………………….. page S2

Chemical Synthesis ……………………………………………………………… page S16

Methods..…………………………...……………………………………………… page S20

Supporting Table………………………………………………………………. page S25

Supporting References……………………………………………………….... page S28

Supporting Figures

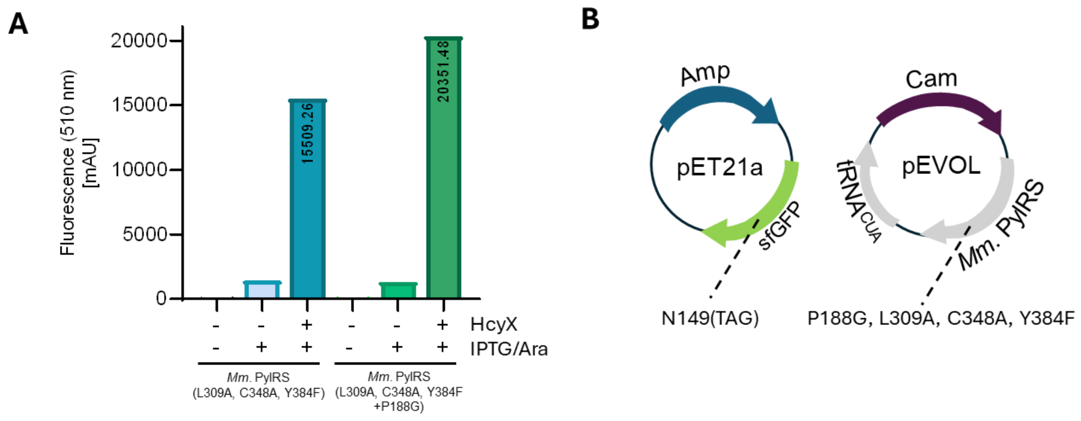

Figure S1: Incorporation efficiency of HcyX. **A)** As described in Figure 2A, sfGFP(N149HcyX)-H_6 (superfolder green fluorescent protein) was expressed in *E. coli* BL21 (DE3) Gold cells by adding **3** (1 mM) to the growth medium. Shown is an analysis of the expression levels by measuring sfGFP fluorescence of the cell cultures at 510 nm (excitation at 488 nm). Fluorescence levels were corrected for OD(600 nm) of the cultures. These data show the positive effect of the P188G mutation[1] on the amber stop codon suppression levels. **B)** Illustration of the vectors used for expression of sfGFP(N149TAG) and incorporation of HcyX at the *amber* position by a *Mm* PylRS mutant. The gene encoding the PylRS mutant is under control of an L(+)-arabinose inducible promotor.

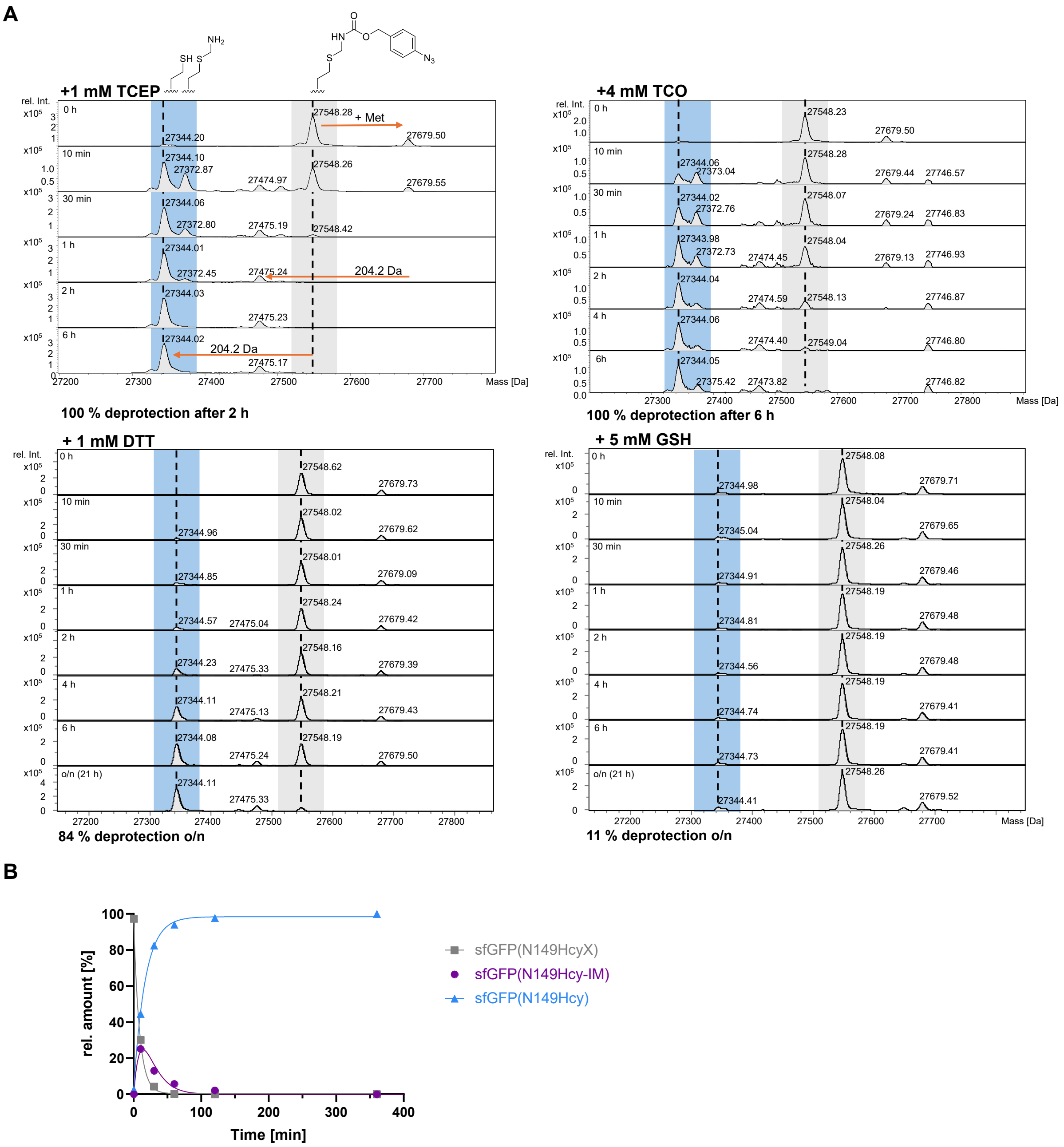

Figure S2: Chemical deprotection of HcyX in sfGFP. **A)** ESI-MS analysis of chemical deprotection of sfGFP(N149HcyX) (10 µM) with 1 mM TCEP, 4 mM TCO-NH_2, 1 mM DTT, and 5 mM GSH, as indicated. Aliquots were removed at specific time points, quenched by addition of 4-azidoaniline and acidified by formic acid (FA). Deprotection of the HcyX side chain led to the temporary accumulation of the aminomethyl intermediate, suggesting its decomposition is a rate-limiting step. **B)** Shown is a plot of the deprotection reaction using 1 mM TCEP, also highlighting the transient appearance of the aminomethyl intermediate (Hcy-IM).

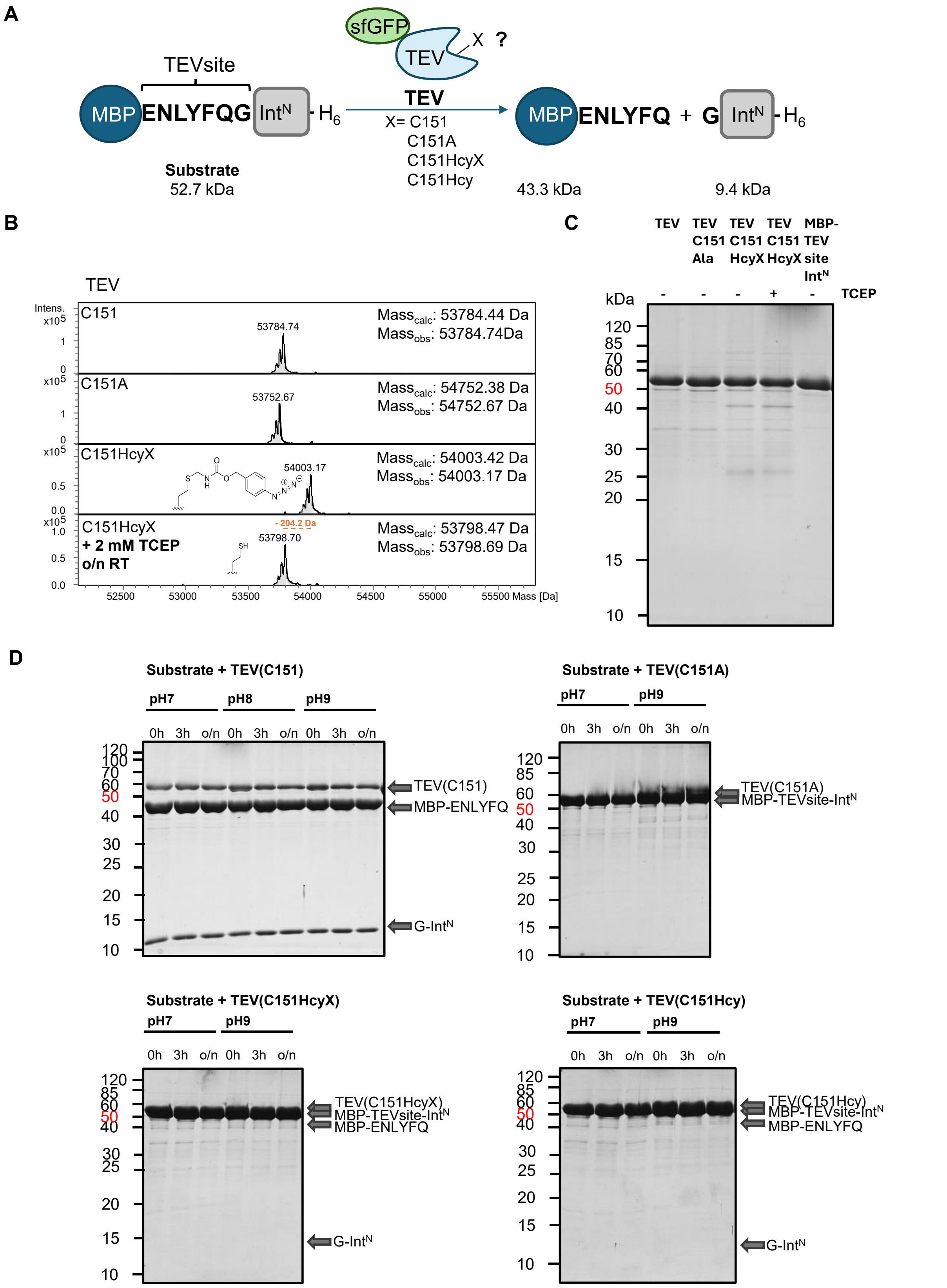

Figure S3: Probing TEV protease suppression with HcyX at Cys151. **A)** Scheme of the assay to monitor proteolytic cleavage of mutants of sfGFP-TEV fusion protein with a model substrate protein harboring the TEV cleavage site ENLYFQ/G. **B)** ESI-MS analysis of sfGFP-TEV mutants. **C)** Coomassie-stained SDS-PAGE gel analysis of purified sfGFP-TEV mutants as analyzed in B). **D)** Coomassie-stained SDS-PAGE gel analyses of the proteolytic cleavage reactions at different pH values. Note that substrate and TEV proteins exhibit similar molecular weights and are therefore not sufficiently separated on these gels.

A

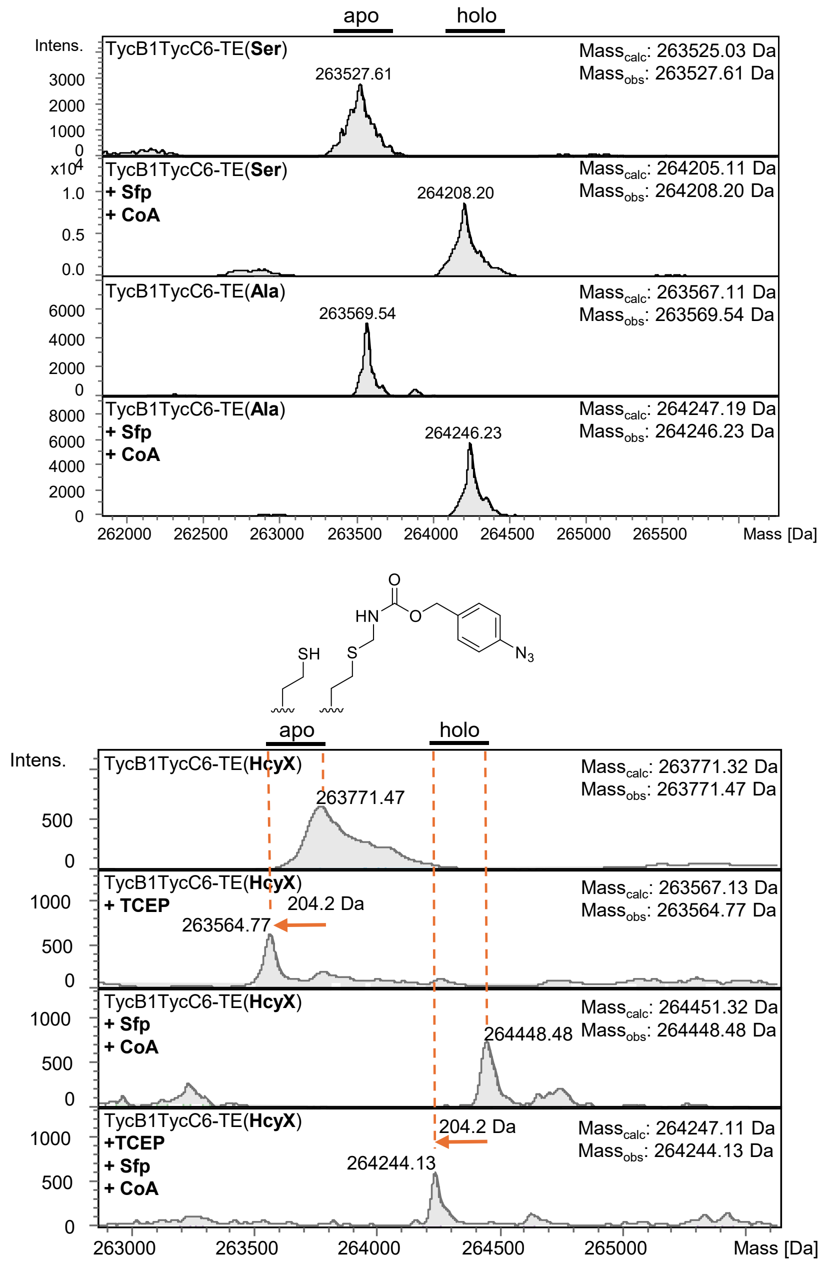

B

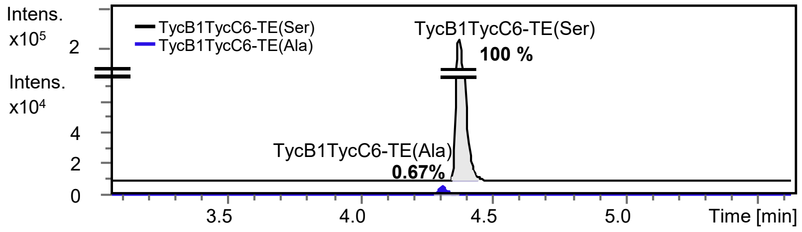

Figure S4: ESI-MS analyses of NRPS proteins and assays. **A)** ESI-MS spectra of TycB1TycC6-TE mutants at the catalytic S2168 of the TE domain (each 10 µM), before (apo form) and after (holo form) 4'-phosphopantetheinylation with Sfp and CoA. For the S2168HcyX mutant also the analysis of deprotection to the S2168Hcy form using TCEP is shown. **B)** Analysis of tripeptide formation (D-Phe-Pro-Leu) from TycA and the Ala mutant TycB1TycC6-TE(S2168A) compared to the assay with the unmutated TE domain. Shown are EIC traces (extracted ion chromatography) of an LC-MS analysis.

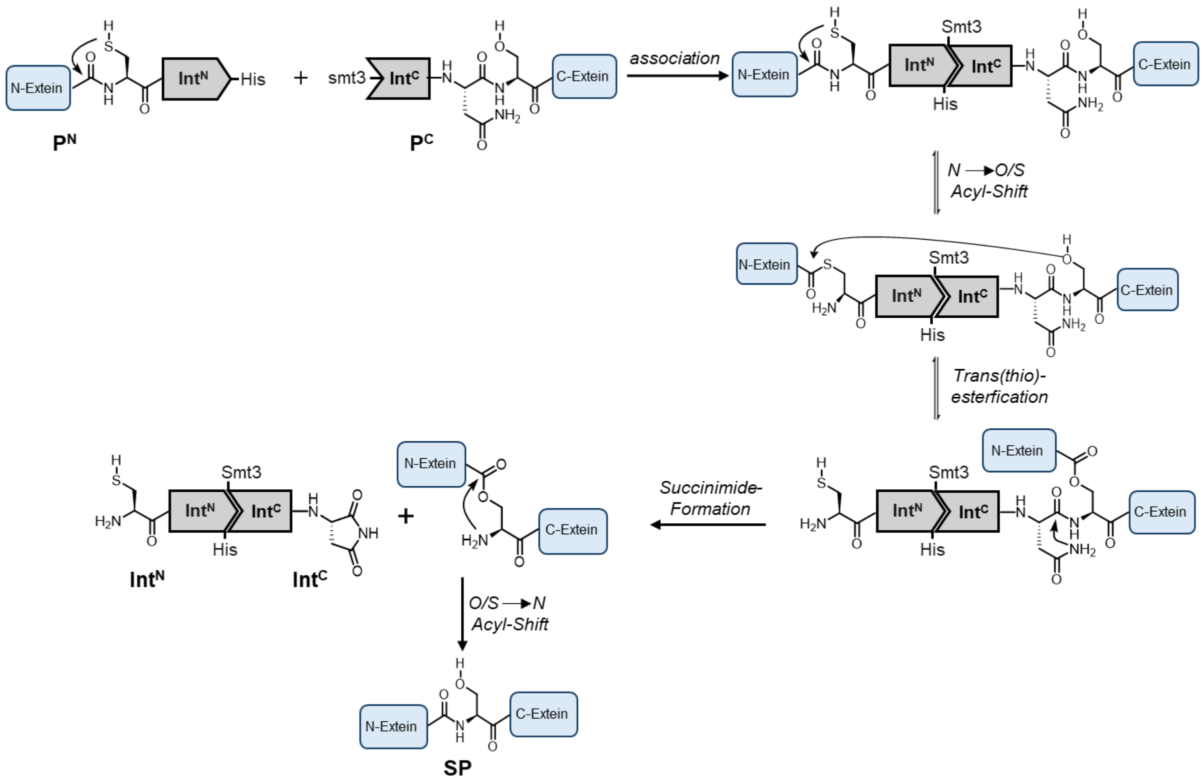

Figure S5: Additional information to protein *trans*-splicing of the Gp41-1 intein. Full mechanism of protein *trans*-splicing shown for the Gp41-1 constructs used in this study. The Gp41-1 intein is a class I intein[2] employing Cys1 and Ser+1 residues at the upstream and downstream splice junctions.

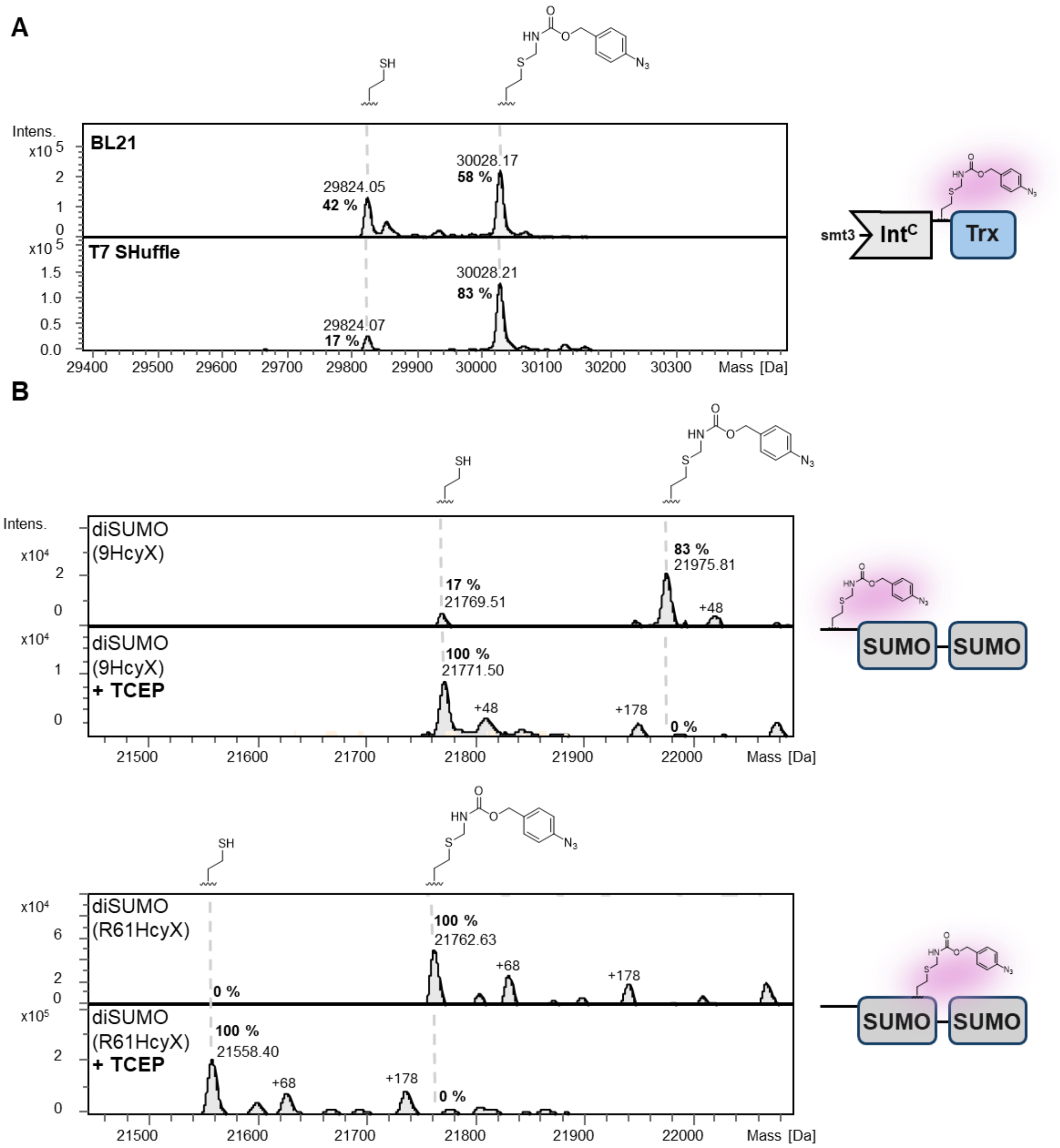

Figure S6: ESI-MS analysis of proteins with HcyX deprotected to Hcy, either *in vivo* or *in vitro*. **A)** ESI-MS spectra of purified Smt3-Int^{C}-Trx produced in *E. coli* BL21 DE3 Gold or *E. coli* T7 Shuffle express. Proteins were purified in the absence of reducing agents or TCO-NH_2 before analysis by mass spectrometry (see Methods). The observed fraction of Hcy-containing protein thus stems from partial *in cellulo* deprotection. **B)** ESI-MS spectra of purified diSUMO proteins before and after TCEP-mediated deprotection suppressed with HcyX at positions 9 and 61 in *E. coli* BL21 DE3 Gold. Proteins with an N-terminal His-tag partially give rise to covalent modifications (e.g. + 178 Da) in *E. coli* due to α-N-gluconoylation, as marked in the spectra.[3-4]

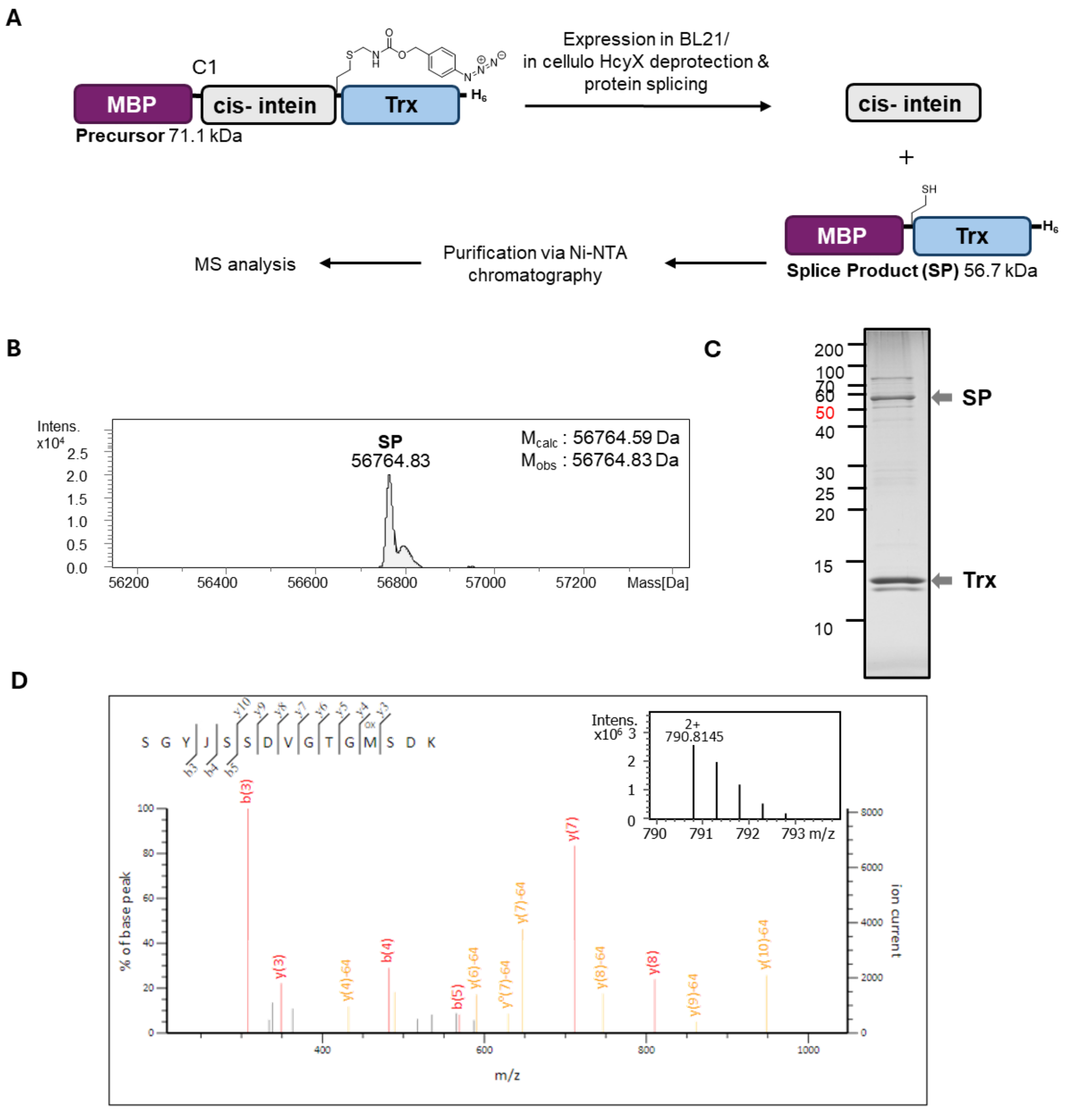

Figure S7: Analysis of protein splicing of the fused *cis*-GP41-1 intein. **A)** Scheme of analysis of *in cellulo* protein splicing of MBP-*cis*Gp41-1(S+1HcyX)-Trx-His. Partial intracellular deprotection of HcyX to Hcy results in the active intein. **B)** ESI-MS analysis of the protein fraction purified by Ni-NTA chromatography **C)** SDS-PAGE analysis of the protein fraction purified by Ni-NTA chromatography (and analyzed by ESI-MS in B)). Shown is a Coomassie-stained gel. **D)** Tandem MS (MS^2) analysis of the purified splice product (SP) MBP-Hcy-Trx-His as shown in B) and C). The letter J represents carbamidoylated Hcy resulting from treatment with iodoacetamide. The inset shows the MS^1 spectrum of the precursor ion. This data confirms the covalent connectivity of the peptide sequences flanking the intein after splicing, including the Hcy+1 residue.

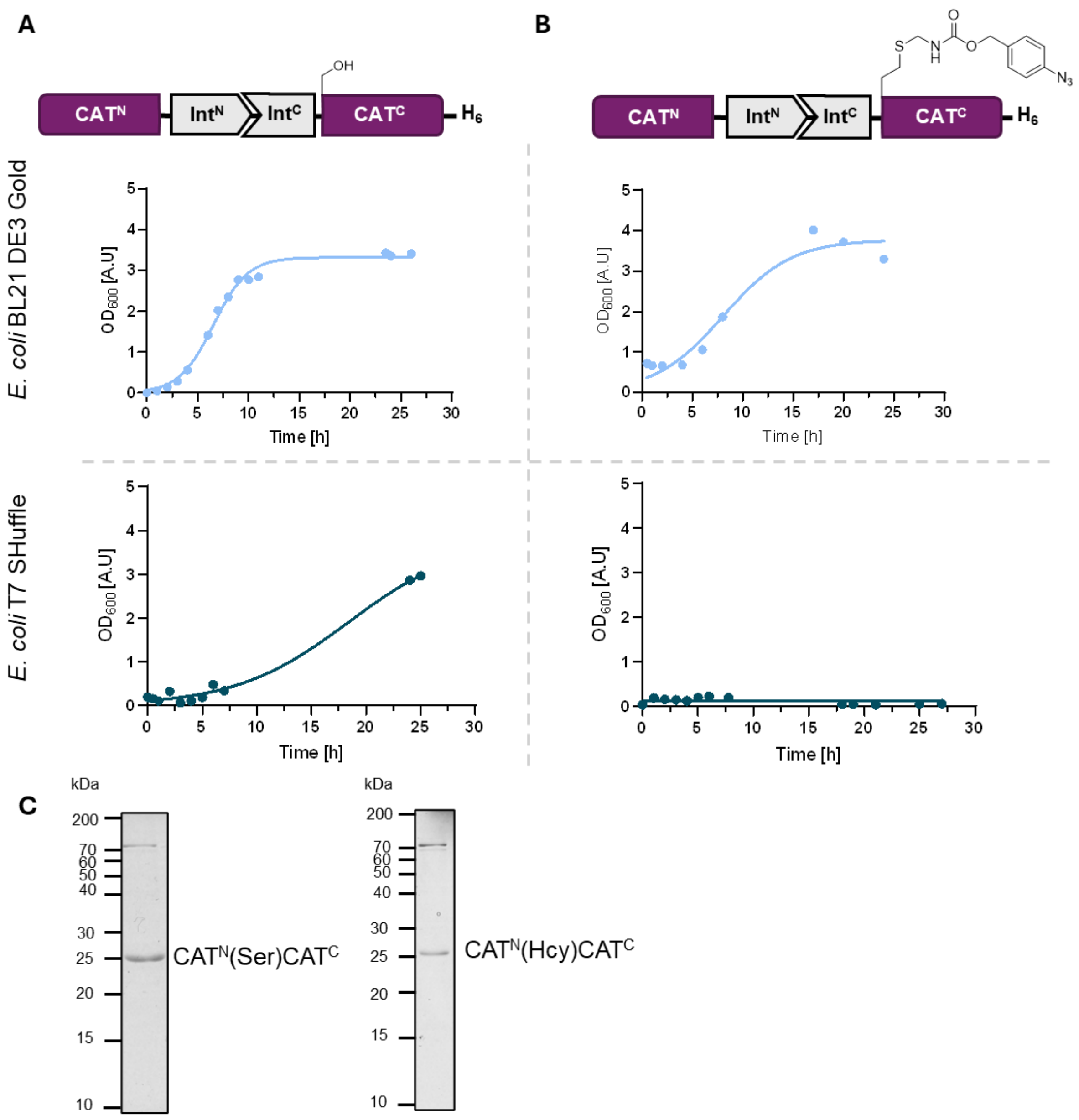

Figure S8: Intein-mediated CAT reconstitution and *E. coli* growth in selective medium containing chloramphenicol (Cam). **A)** The fused *cis*-Gp41-1(S+1) intein with the native S+1 residue inserted in the CAT-cassette was expressed in *E. coli* BL21 DE3 Gold (top) and *E. coli* T7 Shuffle (panel) at 37 °C in presence of Cam^{50} (chloramphenicol at 50 µg/mL) and cell growth was monitored over time (OD at 600 nm), following inoculation from an overnight pre-culture (without Cam^{50}). **B)** Same as in A) but using the *cis*-Gp41-1(S+1HcyX) intein, and with added HcyX (1 mM) in both the overnight pre-culture (without Cam^{50}) and the monitored culture (with Cam^{50}). These data show that sufficient *in cellulo* deprotection of HcyX to Hcy to support CAT reconstitution at levels required to rescue cell growth only occurred in the BL21 and not in the T7 SHuffle strain. **C)** SDS-PAGE analyses of purified CAT^N(Ser)CAT^C and CAT^N(Hcy)CAT^C from *E. coli* BL21 DE3 Gold. Shown are Coomassie-stained gels. These protein samples were used for the ESI-MS analysis shown in Figure 6.

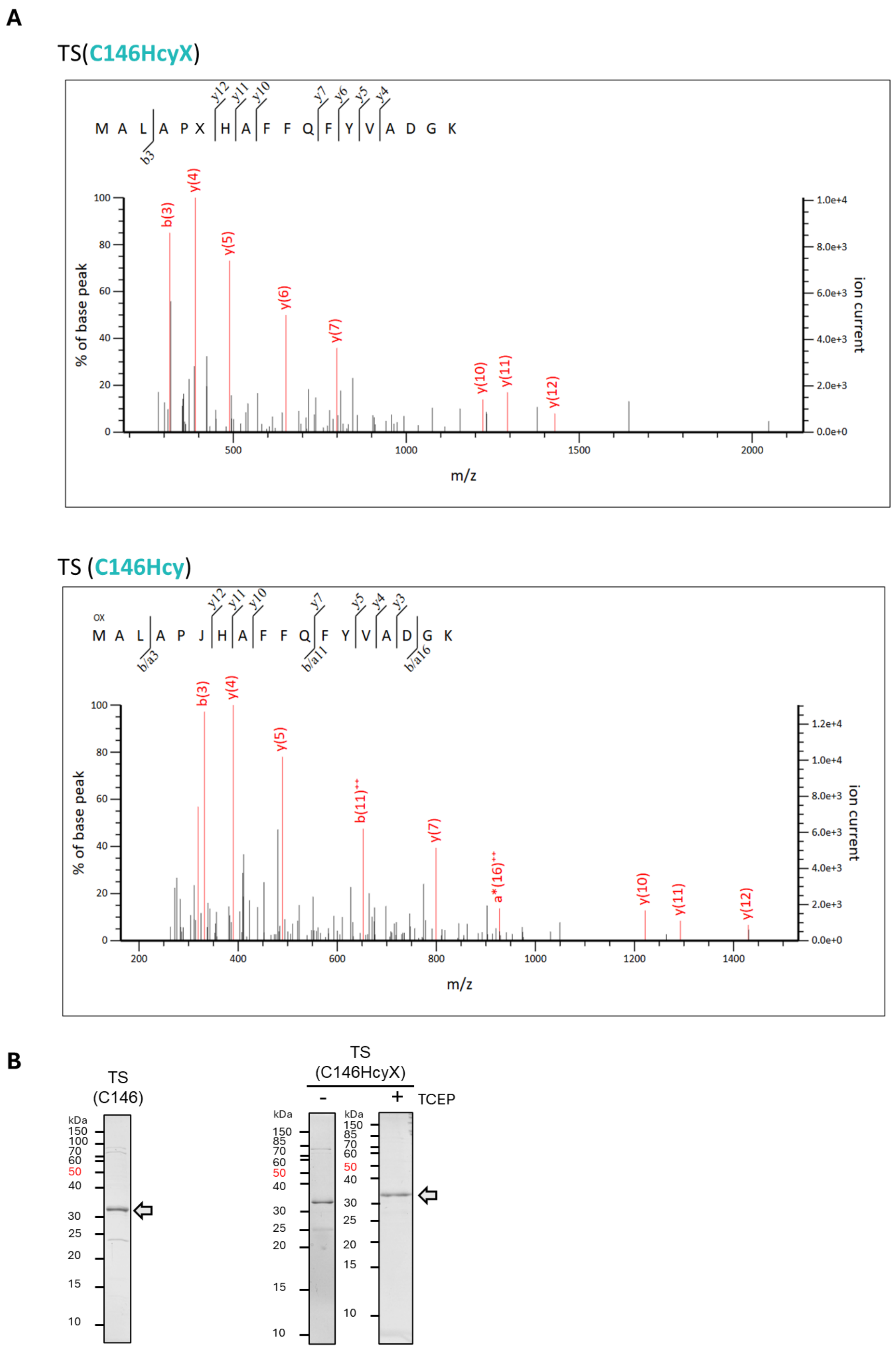

Figure S9: Analyses of purified thymidylate synthase (TS) constructs. **A)** Tandem MS analysis (MS^2) of TS(C146HcyX) and ThyA(C146Hcy). Shown are the spectra representing the tryptic peptide fragment containing the position 146 to prove HcyX incorporation and deprotection, respectively. **B)** SDS-PAGE analyses of the indicated, purified TS mutants using for the MS analysis shown in A) and in Figure 7C. J = carbamidoylated Hcy resulting from reaction with iodoacetamide; X = HcyX.

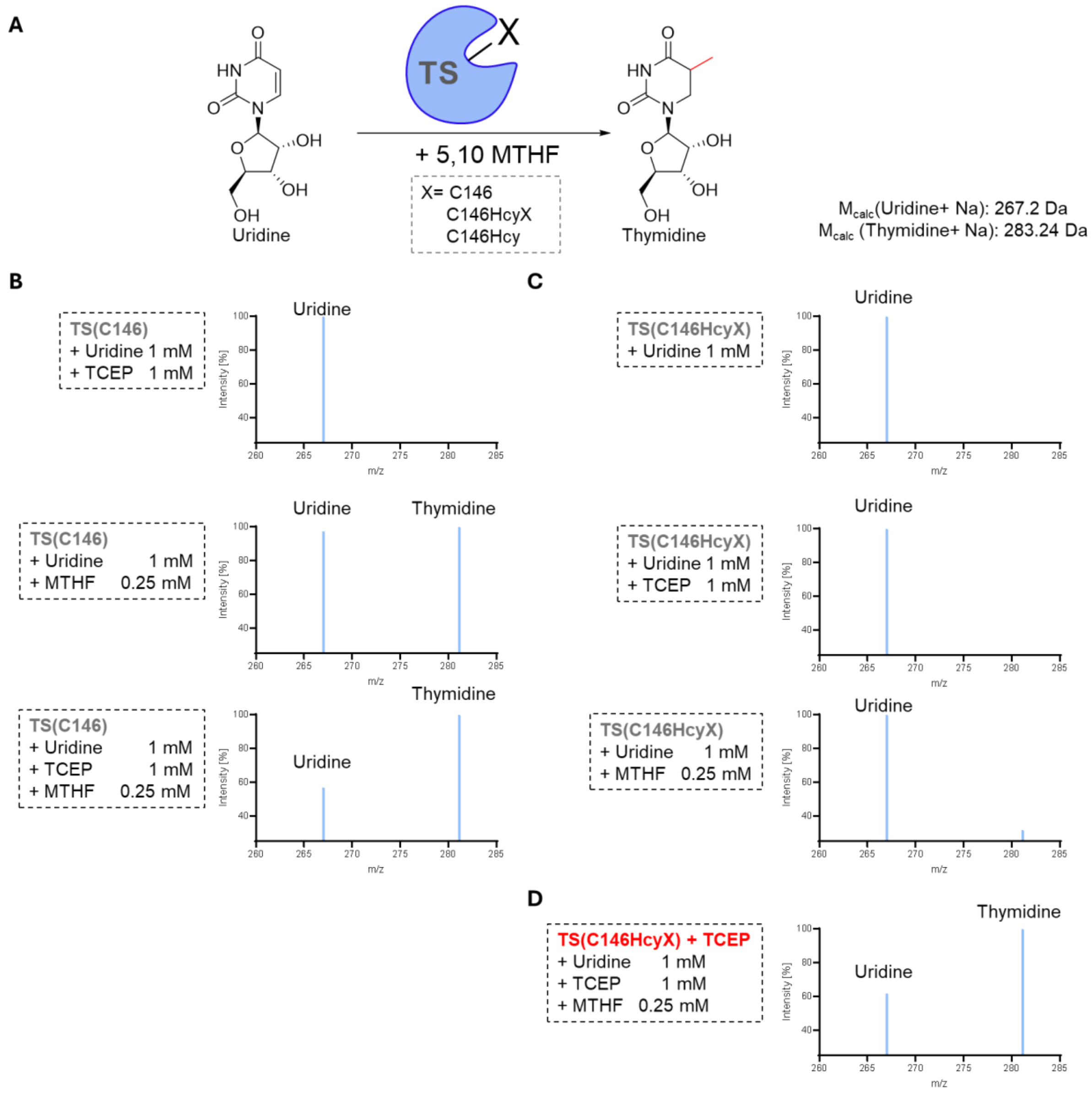

Figure S10: Biochemical analysis of thymidylate synthase (TS) mutants. **A)** Reaction scheme of uridine into thymidine conversion. **B)** LC-MS analyses of thymidine formation by the wildtype TS(C146) using various substrate and cofactor (5,10-MTHF) combinations and TCEP controls. **C)** Same es in B) but using the protected TS(C146HcyX) mutant. **D)** Same as in B) but using TS(C146HcyX) pre-treated with TCEP to convert it into the deprotected TS(C146Hcy) mutant. The LC-MS analyses in B) to D) were performed using a C18-column. Shown are the MS data from single ion monitoring (SIM) mode to record the masses corresponding to uridine and thymidine.

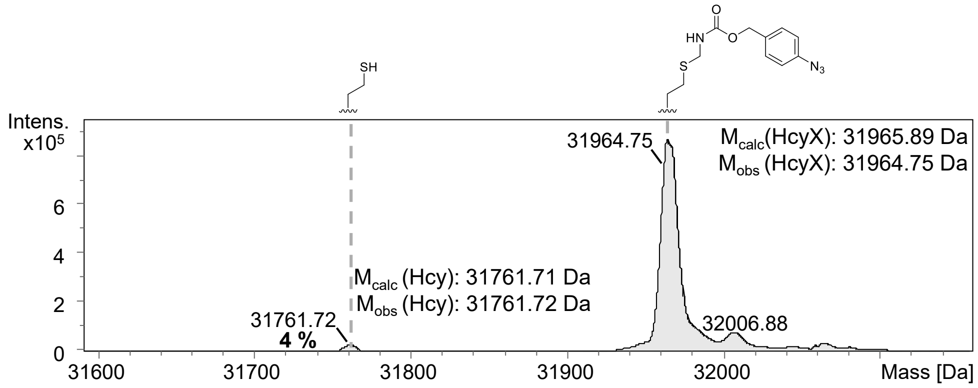

Figure S11: Analysis of TS(C146HcyX) *in cellulo* deprotection. Shown is an ESI-MS analysis of TS(C146HcyX) purified from *E. coli* Δ*thyA* cells expressing *thyA*(C146TAG) overnight in the presence of HcyX (1 mM) at 37 °C.

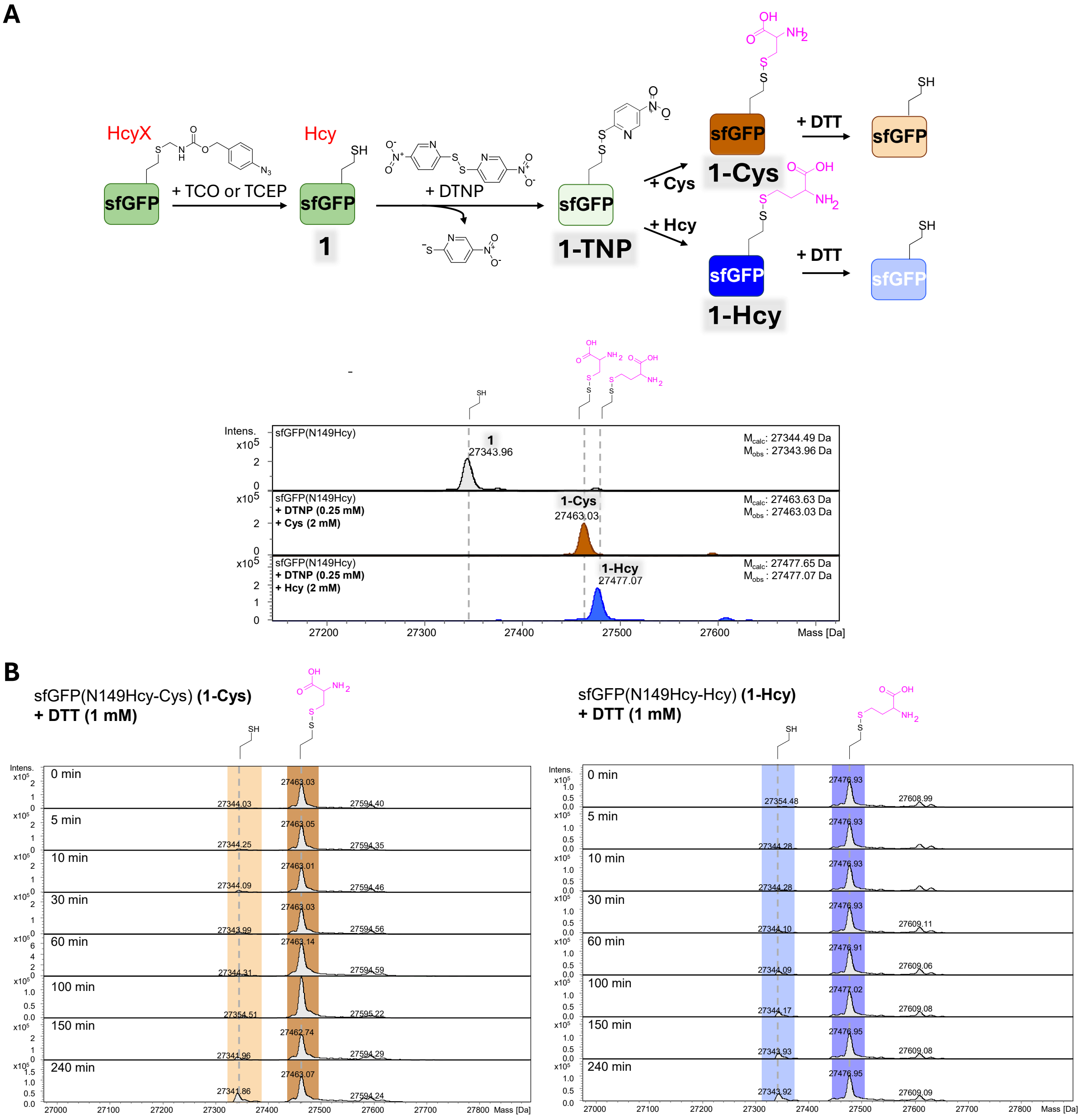

>>>>> Figure S12 continued on next page

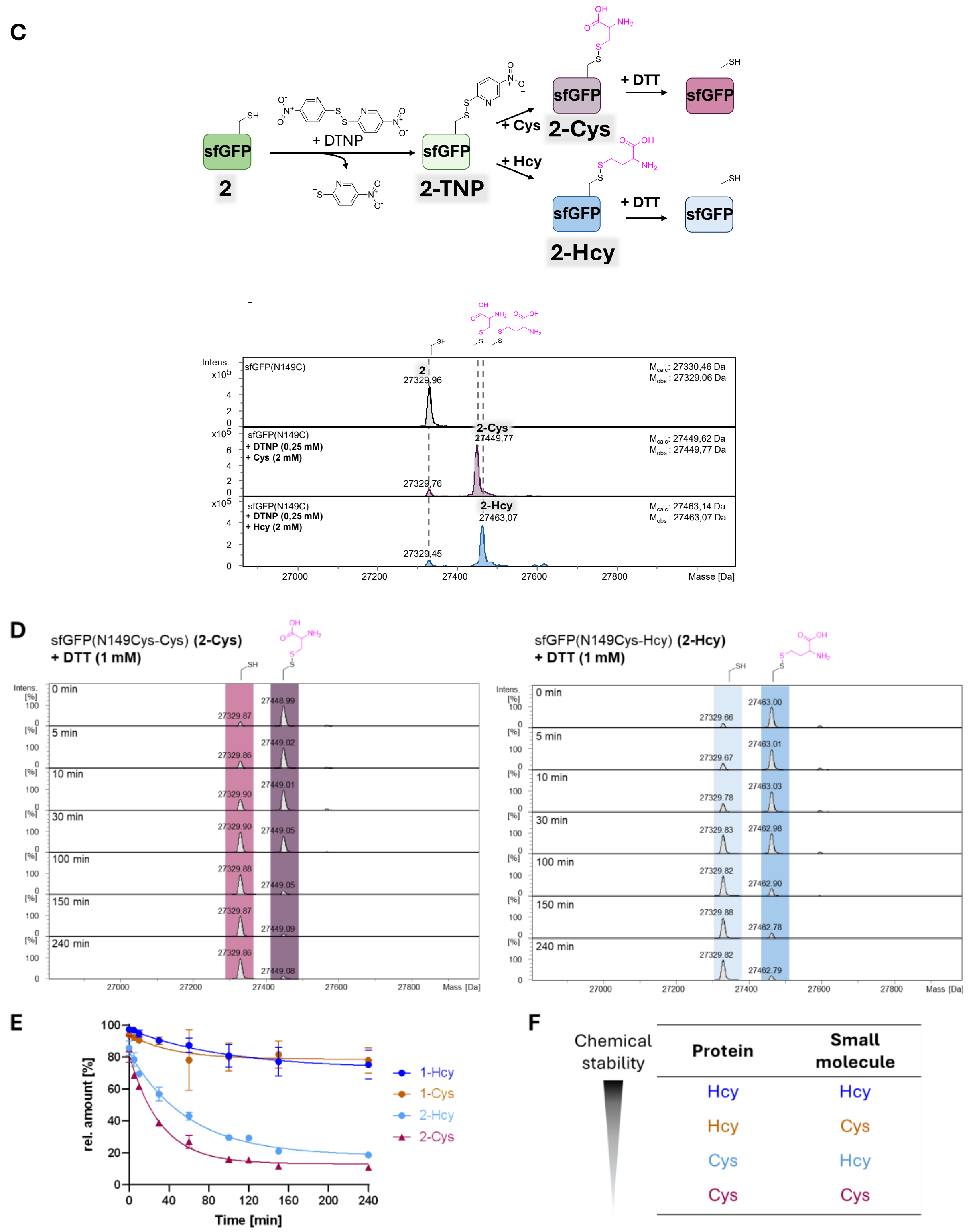

Figure S12: Generation of Hcy-based disulfides and analysis of their chemical stability. All possible combinations of disulfide bonds between a protein (sfGFP with Hcy or cysteine) and a small molecule (Hcy or cysteine) were generated and analyzed for their reaction with excess free thiol (DTT). **A)** Scheme of the generation of the Cys- and Hcy- disulfides based on sfGFP(N149Hcy) (top panel) and their verification by ESI-MS analysis (bottom panel). **B)** ESI-MS analysis of disulfide reduction by DTT (1 mM) of sfGFP(N149Hcy-Cys) (**1-Cys**) (left panel) and sfGFP(N149Hcy-Hcy) (**1-Hcy**) (right panel) over time. Both proteins were used at 10 µM. **C)** Scheme of the generation of the Cys- and Hcy- disulfides based on sfGFP(N149C) (top panel) and their verification by ESI-MS analysis (bottom panel). **D)** ESI-MS analysis of disulfide reduction by DTT (1 mM) of sfGFP(N149Cys-Cys) (**2-Cys**) (left panel) and

sfGFP(N149Cys-Hcy) (**2-Hcy**) (right panel) over time. Both proteins were used at 10 µM. **E)** Diagram of the disulfide cleavage reactions as monitored in B) and D). **F)** Overview of the determined chemical stability of the four different disulfide bonds against reduction with DTT. All experiments were performed in duplicate (n=2). The error bars represent mean standard deviations.

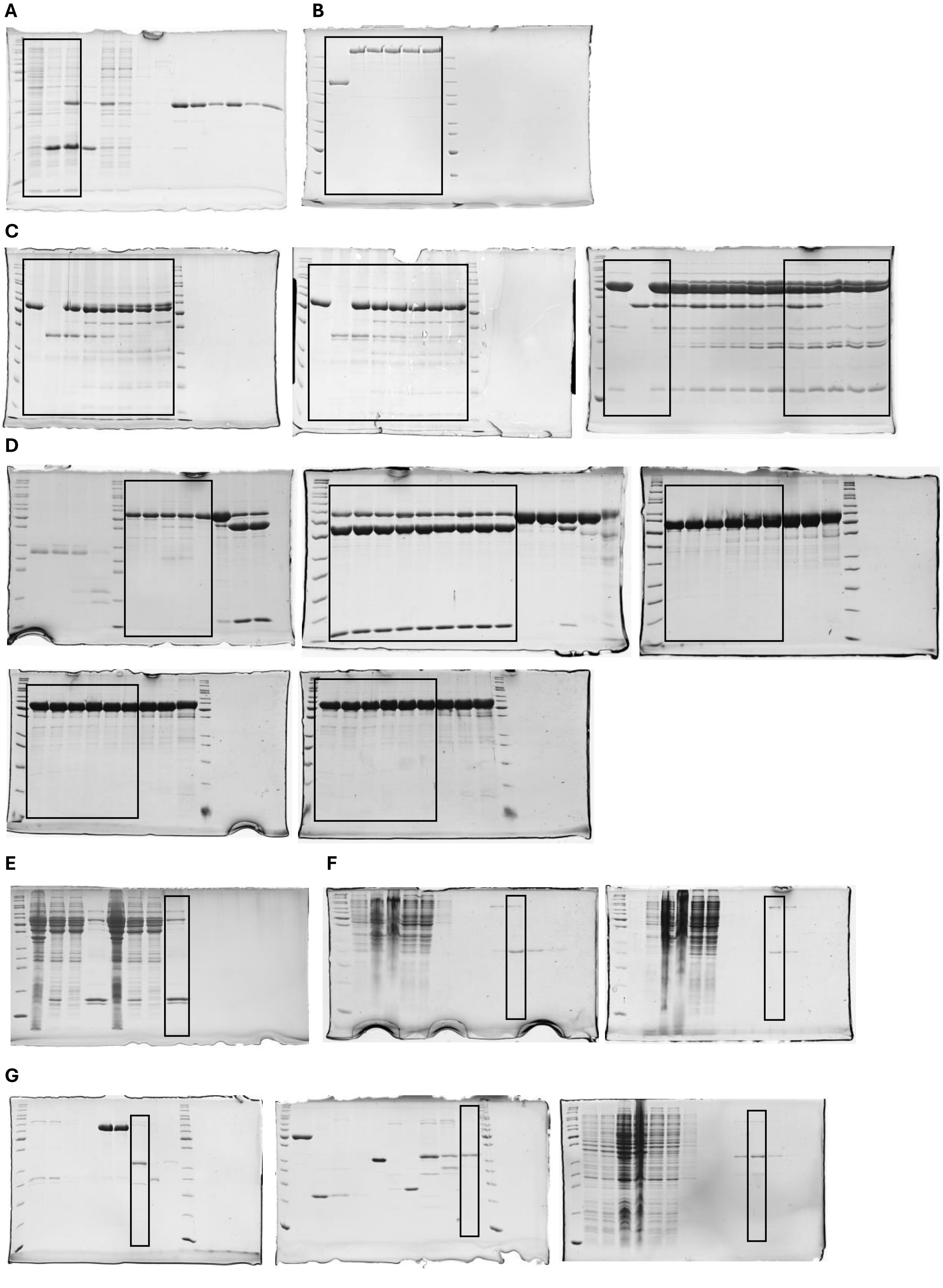

Figure S13: Collection of all uncropped SDS-PAGE gels. The black frame indicates the cropped section used in the final images. **A)** Figure 2. **B)** Figure 3. **C)** Figure 4. **D)** Figure S2. **E)** Figure S7. **F)** Figure S8. **G)** Figure S9.

General

Chemicals used in this study were purchased from Activate Scientific, Merck, Sigma Aldrich, Carl Roth, Chem Shuttle, Fisher Scientific and Chem Pur. Oligonucleotides were purchased from Biolegio. All DNA constructs were verified by DNA-Sequencing (Microsynth Seqlab). Protein mass spectrometry was performed using a MicroTof ESI from Bruker Daltonics. NMR-spectra were recorded using Bruker Avance II 300 and Bruker Avance II 400 spectrometers. Peak multiplicity is represented by following abbreviations: s = singulet, d = doublet, t = triplet, m = multiplet, dd = doublet of doublets, dq = doublet of quartets, td = triplet of doublets. Chemical shifts of NMR spectra were referenced to the solvent signal.

Chemical Synthesis of HcyX (3)

(4-Azidophenyl)methanol

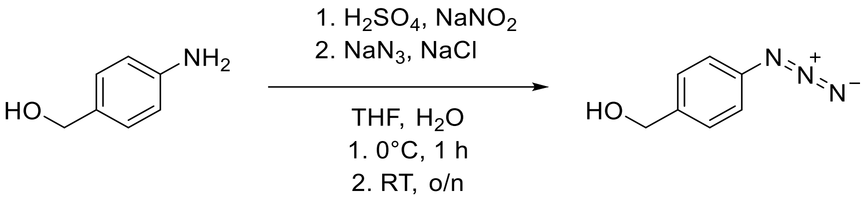

The synthesis was adapted from Gorska *et al.*.[5] 4-Aminobenzylalcohol (4.3 g, 35 mmol, 1 eq.) was dissolved in THF (50 mL) and H_2O (120 mL) was added. The solution was cooled to 0 °C, and concentrated H_2SO_4 (9.6 mL) and $NaNO_2$ (2.9 g, 42 mmol, 1,2 eq) in H_2O (20 mL) were added dropwise. The solution was stirred for 1 h at 0°C. Subsequently, NaN_3 (2.72 g, 42 mmol, 1.2 eq.) in H_2O (10 mL) was added dropwise and the approach was stirred overnight, while warming to room temperature. The solution was quenched with sodium chloride and extracted with DCM. The combined organic phases were dried over sodium sulphate, filtered and concentrated under reduced pressure. The crude product was purified by silica chromatography (EtOAc/cyclohexane, 1:5). The product was obtained with a yield of 93 % as a yellow powder.

^{1}H NMR (400 MHz, $CDCl_3$):δ = 7.40 – 7.32 (m, 1H), 7.06 – 6.98 (m, 1H), 4.67 (s, 1H).

(4-Azidobenzyl)carbamate

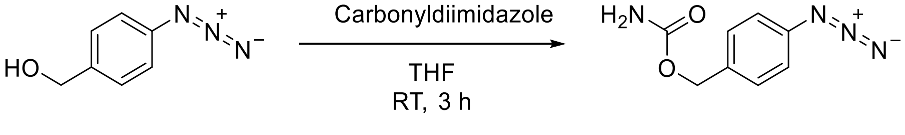

The reaction procedure was adapted from Choi *et al.*.[6] 4-Azidobenzylalcohol (4.87 g, 32.5 mmol, 1 eq.), carbonyl diimidazole (CDI) (7.9 g, 48.75 mmol, 1.5 eq.) and THF (60 mL) were combined and stirred for 3 h at room temperature under Ar. NH_4OH (20 mL) was added and the approach was stirred for further 2 h. The reaction mix was washed with HCl (1 M) and

extracted with EtOAc. The combined organic phases were dried over sodium sulphate, filtered and concentrated under reduced pressure. The crude product was purified by silica chromatography (EtOAc/cyclohexane, 1:1) and the product was obtained with a yield of 90 % as yellow solid.

^{1}H NMR (400 MHz, $CDCl_3$): δ 7.44 – 7.32 (m, 1H), 7.06 – 6.98 (m, 1H), 5.06 (s, 1H), 4.66 (s, 1H).

4-Azidobenzyl (hydroxymethyl)carbamate

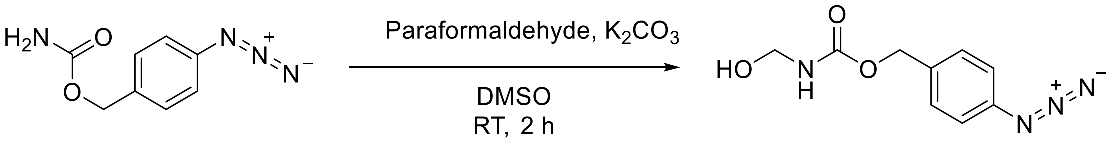

The synthesis was adapted from Uprety *et al.*.[7] To a solution of the carbamate-intermediate (2 g, 1.04 mmol, 1 eq.) in dry DMSO (25 mL) were added paraformaldehyde (0.43 g, 1.14 mmol, 1.1 eq.) and K_2CO_3 (0.17 g, 0.12 mmol, 0.12 eq) under Ar at room temperature. It was stirred for 2 h at room temperature. The mixture was extracted by EtOAc and washed with sodium chloride. The combined organic phases were dried over sodium sulphate, filtered and concentrated under reduced pressure. The crude product was purified by silica chromatography (acetone/DCM, 1:7) and the product was obtained with 45 % yield as a yellow oil.

^{1}H NMR (400 MHz, $CDCl_3$): δ = 7.39 – 7.31 (m, 1H), 7.06 – 6.98 (m, 1H), 5.69 (s, 1H), 5.10 (s, 1H), 4.74 (t, J = 7.3 Hz, 1H), 2.93 (t, J = 7.7 Hz, 1H).

HRMS (ESI+): $[M+Na]^+$ m/z_{obs}= 245.0 ; m/z_{calc}= 245.1

Methyl (tert-butoxycarbonyl)-L-Homocysteinate

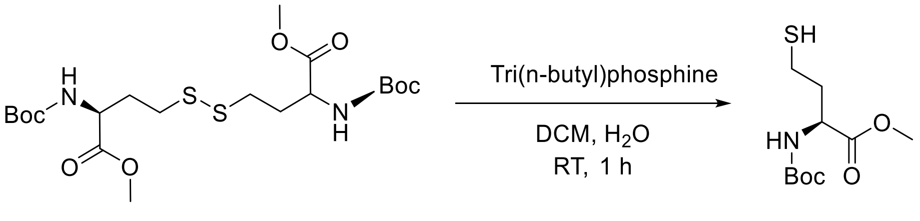

The reaction procedure was adapted from Lherbert *et al.*.[8]. *N*-Boc-L-Homocystine methylester (1 g, 2.01 mmol, 1 eq.) was dissolved in DCM (8.8 mL) under Ar. H_2O (0.5 mL) and tri-(n-butyl)phosphine (0.55 mL, 2.21 mmol, 1.1 eq.) were added and the mixture stirred for 1 h at room temperature. The organic phases were isolated, dried over sodium sulphate, filtered and concentrated under reduced pressure. The crude product was purified by silica chromatography (10-20% EtOAc/cyclohexane) and the product was obtained as a colorless oil with a yield of 80 %.

^{1}H NMR (300 MHz, $CDCl_3$): δ = 5.01 (d, J = 7.9 Hz, 1H), 4.40 (q, J = 7.5 Hz, 1H), 3.68 (d, J = 6.8 Hz, 1H), 2.61 – 2.42 (m, 1H), 2.04 (dq, J = 13.4, 7.1 Hz, 1H), 1.86 (dtd, J = 14.0, 8.2, 6.3 Hz, 1H), 1.59 – 1.47 (m, 1H), 1.38 (s, 1H).

HRMS (ESI+): $[M\text{-}Boc+H]^+$ m/z_{obs}= 150.1 ; m/z_{calc}= 150.10

Mehtyl *S*- (((((4-azidobenzyl)oxy)carbonyl)amino)methyl)- *N*- (tert-butoxycarbonyl) Homocysteinate

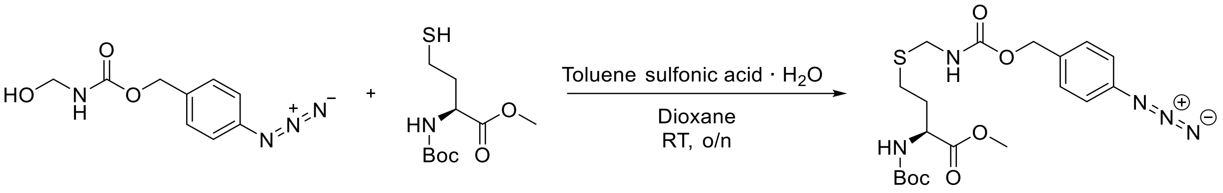

The synthesis reaction procedure was adapted from Uprety *et al.*.[7] *N*-boc-L-homocysteine methylester (1.07 g, 4.31 mmol, 1 eq.) was dissolved in dry dioxane under Ar. 4-azidobenzyl (hydroxymethyl)carbamate (1.44 g, 6.47 mmol, 1.5 eq.) and toluene sulfonic acid monohydrate (66 mg, 0.35 mmol, 0.08 eq) were added and the mixture was stirred over night at room temperature. Extraction with EtOAc, washing of combined of organic phases with $NaHCO_3$, drying over sodium sulphate and volume reduction under reduced pressure gave the crude product. It was purified by silica chromatography (10-50% EtOAc/cyclohexane) and the product was obtained with a yield of 68 %.

^{1}H NMR (300 MHz, $CDCl_3$): δ = 7.37 (t, *J* = 9.3 Hz, 1H), 7.10 – 7.00 (m, 1H), 5.34 (s, 1H), 5.18 – 5.06 (m, 1H), 4.47 – 4.28 (m, 1H), 3.77 (s, 1H), 2.66 (m, 1H), 2.09 – 1.94 (m, 1H), 1.46 (s, 1H).

HRMS (ESI+): $[M\text{-}Boc+H]^+$ m/z_{obs}= 354.00 ; m/z_{calc}= 453.17

S-(((((4-Azidobenzyl)oxy)carbonyl)amino)methyl)-*N*-(tert-butoxycarbonyl) Homocysteine

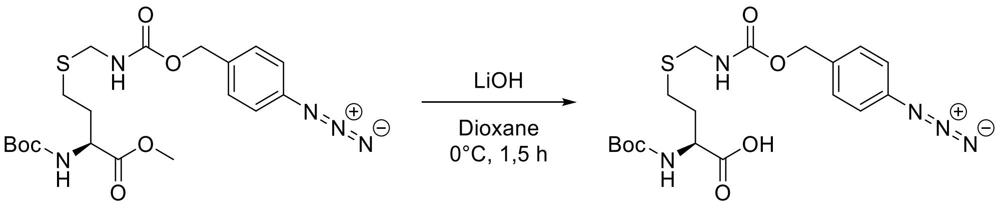

The synthesis reaction procedure was adapted from Uprety *et al.*.[7] Methyl *S*-(((((4-azidobenzyl)oxy)carbonyl)amino)methyl)-*N*-(*tert*-butoxycarbonyl) homocysteinate (0.823 g, 1.82 mmol, 1 eq) was dissolved in dry dioxane (20 mL) under Ar and brought to 0 °C. LiOH 2 M in H_2O (20 mL) was added and stirred for 1.5 h at 0 °C. The reaction mixture was diluted with H_2O and washed with diethyl ether. The aqueous phase was acidified by HCl (1 M) to pH 2-3 and extracted with EtOAc. The product was obtained with a yield of 98 %.

^{1}H NMR (400 MHz, $CDCl_3$): δ = 7.35 (dd, *J* = 8.6, 2.3 Hz, 1H), 7.05 – 6.98 (m, 1H), 5.48 (s, 1H), 5.36 (s, 1H), 5.16 – 5.04 (m, 1H), 4.45 – 4.21 (m, 1H), 2.76 – 2.56 (m, 1H), 2.21 (s, 1H), 2.05 (s, 1H), 1.44 (s, 1H).

HRMS (ESI+): $[M\text{-}Boc+H]^+$ m/z_{obs}= 340.10 ; m/z_{calc}= 439.10

S-(((((4-Azidobenzyl)oxy)carbonyl)amino)methyl) homocysteine

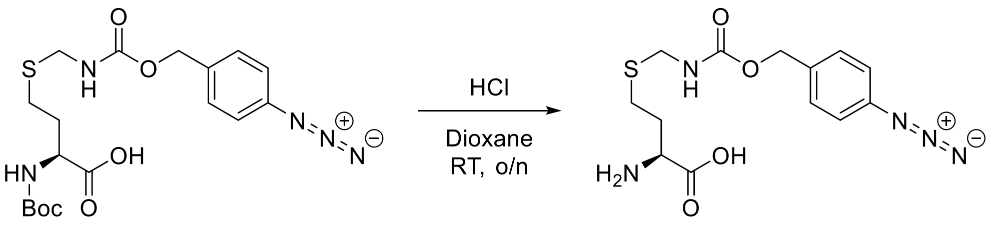

The synthesis reaction procedure was adapted from Uprety *et al.*.[7] *S*-(((((4-azidobenzyl)oxy)carbonyl)amino)mehtyl)-*N*-(*tert*-butoxycarbonyl)homocysteine (222 mg, 0.51 mmol, 1 eq) was dissolved in dry dioxane (6 mL) under Ar and HCl in dioxane (4 M, 1 mL) was added. The approach was stirred over night at room temperature. Dioxane was diluted by DCM and coevaporated under reduced pressure. Coevaporation was repeated three times and the residual was dissolved in MeOH (5 mL). For precipitation the solution was added dropwise into ice cold diethyl ether, while a final volume of 10 % MeOH (v/v) was not exceeded. Centrifugation of the suspension at 12000 rpm for five minutes at 4 °C, decanting and resuspension in fresh, ice-cold diethyl ether were repeated three times. The product was obtained with a yield of 90 %.

^{1}H NMR (400 MHz, DMSO-d6): δ = 8.45 (s, 5H), 8.06 (t, *J* = 6.4 Hz, 1H), 7.48 – 7.38 (m, 3H), 7.20 – 7.10 (m, 3H), 5.03 (s, 2H), 4.65 – 4.52 (m, 1H), 4.21 (m, 3H), 4.00 – 3.81 (m, 2H), 2.72 (m, 3H), 2.08 (m, 3H).

HRMS (ESI+): $[M+H]^+$ m/z_{obs}= 340.10 ; m/z_{calc}= 339.10

^{1}H NMR spectrum of HcyX (**3**) HCl salt (DMSO-d6)

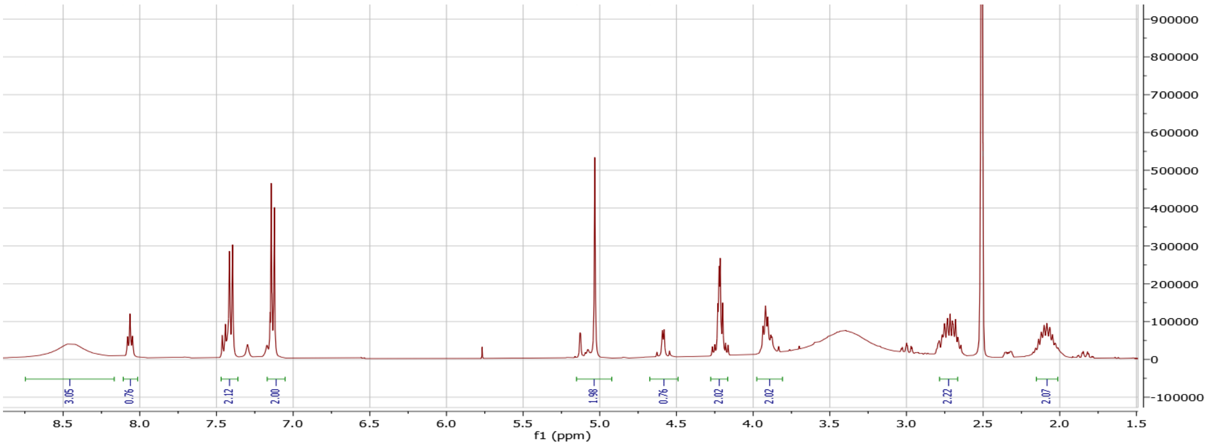

Methods

Expression and affinity chromatography of recombinant proteins

For expression of recombinant proteins without an unnatural amino acid, *E. coli* BL21 DE3 Gold or *E. coli* T7 SHuffle were transformed with the respective plasmid (see Table S1) and grown in LB medium containing antibiotics (ampicillin 100 µg/mL; kanamycin 50 µg/mL; or chloramphenicol 34 µg/mL). The overnight cultures were inoculated in LB-medium with volumes of 300 mL or 600 mL containing antibiotics. Cultures were induced by addition of L(+)-arabinose (0.2 % w/v) or IPTG (0.4 mM) at an OD_{600nm} of 0.6-1.0 and shaken for 4 h (sfGFP, diSUMO, split intein, TEV protease) or overnight (Cam-cassette, thymidylate synthase) at 28 or 37 °C.

For expression of proteins with the unnatural amino acid, *E. coli* BL21 DE3 Gold or *E. coli* T7 Shuffle were cotransformed with a plasmid containing the gene of POI with an *amber* codon (Table S1) and one coding for the *Mm.* PylRS(P188G, L309A, C348A, Y384F)/tRNA pair. The respective mutations were introduced by site-directed mutagenesis into a plasmid with *Mm.* PylRS(N346A, C348A) kindly provided by Wenshe Liu.[9] The mutations were previously reported in works of Ge *et al.* with *Mb*. PylRS(L274A, C313S, Y349F),[10] Wesalo *et al*. with *Mb*. PylRS(L274A, C313A, Y349F),[11] and Cho *et al*. with PylRS(P188G).[1] Overnight cultures in lysogeny broth (LB) medium containing antibiotics (ampicillin 100 µg/mL, kanamycin 50 µg/mL or chloramphenicol 34 µg/mL) were inoculated in LB- medium of 50 mL at 37 °C until an OD_{600} of 0.6-1.0 was reached. Cultures were induced by addition of L(+)-arabinose (0.2 % w/v) and IPTG (0.4 mM), while the ncAA HcyX 1 mM was added. The cultures were shaken for 4 h (sfGFP, diSUMO, split intein, TEV protease) or overnight (NRPS, Cam-cassette, thymidylate synthase) at 37 °C.

Cells were then harvested by centrifugation (20 min, JA10 2831 g, JA14 2455 g, 4 °C) and resuspended in Ni-NTA buffer (50 mM Tris, 300 mM NaCl, pH 8). Cells were lysed by sonication (Sonopuls, Bandelin) for up to 12 min (pulse 10 sec on / 15 sec off, amplitude 60 %) and cell debris was removed by centrifugation (30 min, JA14 22095 g, 4 °C). The supernatant was purified by Ni-NTA affinity chromatography (Ni-NTA resins from Cube Biotech). The bound protein fraction was washed with 30 mM imidazole in Ni-NTA buffer three times. The His-tagged proteins were eluted with 250 mM imidazole and dialyzed in PBS (pH 7.4) or in exceptions in an NRPS-assay buffer (pH 7).

The obtained purified proteins were flash freezed in liquid nitrogen at – 200 °C and stored at -70 °C in buffer containing 10 % of glycerol.

Cloning of $pEVOL^{KanR}$ encoding PylRS/tRNA pair

To co-express the *Mm*. PylRS/tRNA pair in *E. coli* strains that also expressed a gene for chloramphenicol acetyltransferase (Cam^R gene, encoded in a pDrive derivative with removed Kan^R gene and pMB1 ori) we swapped the antibiotica resistance gene of the pEVOL vector from Cam^R to Kan^R (encoding the aminoglycoside-3'-phosphotransferase APH(3'), PCR-amplified from pET28a). To this end, pEVOL was mutated by a restriction and ligation approach with *Nhe* I and *Xho* I restriction enzymes and the Kan^R gene was inserted. The genes for PylRS and tRNA remained unchanged.

Deprotection of HcyX in proteins (*in vitro*)

Deprotection of the HcyX side chain in proteins was performed by adding either TCEP (1 mM), TCO-NH_2 (4 mM), DTT (1 mM), or GSH (5 mM) to a solution of 10 µM protein(HcyX) in PBS. Incubation was carried out at 20 °C, except for TCO-NH_2-approaches, where the protein was incubated at 37 °C. The reactions were quenched by addition of 4-azidoaniline (5 mM) and by acidifying to pH 1-2 with formic acid (FA). The samples were analyzed by LC-MS.

Mass spectrometry of proteins

The set up for mass spectrometry was an UltiMate™ 3000 RS system (Thermo Fisher Scientific Inc., MA, USA) connected to a maXis II UHR-TOF mass spectrometer (Bruker Daltonik GmbH, Bremen, GE) with a standard ESI source (Apollo, Bruker Daltonik GmbH, Bremen, GE). The native proteins or peptides were acidified to a pH of 1-2 with formic acid (FA), centrifuged (2 min, 12000 rpm, 4 °C) and injected. A C4 column (Advance Bio RP-mAb C4, 2.1 mm x 50 mm, 3.5 µm, Agilent Technologies, Waldbronn, GE) or a C18 column (ZORBAX SB-C18 RR HT, 3 mm x 50 mm, 1.8 µm, Agilent Technologies, Waldbronn, GE) were used at a flow rate of 0.6 mL/min with eluents A and B (eluent A: 0.1% formic acid in H_2O; eluent B: 0.1% formic acid in acetonitrile). The method run a desalting period (7 min, 5% B) and was followed by a gradient (5-60% B in 2 min).

[capillary voltage 4500 V, end-plate offset 500 V, dry temperature 200 °C, mass range m/z 300-3000, nebulizer 3.5 bar, flow rate of dry gas 8.0 L/min]

Analysis of mass spectra was performed by using DataAnalysis 4.4 (Bruker Daltonics GmbH, Bremen, GE).

Tryptic digest of proteins for MS/MS analyses

For tryptic digest of proteins, Coomassie-stained gel bands were destained by shaking in 50 % MeOH/ H_2O/ 0.1 % TFA 400 µL at 40 °C overnight. The supernatant was discarded, the gel was washed twice with H_2O and taken up in 400 µL acetonitrile. After 20 min the supernatant was discarded. The gel was dried in the speedvac (15 min, 60 °C). Reduction was performed by addition of DTT (40 µL, 10 mM) dissolved in NH_4HCO_3 (100 mM) at 56 °C for 20 min. Afterwards the gel was washed with 400 µL buffer NH_4HCO_3 (100 mM) and the supernatant was again discarded. Alkylation was carried out by addition of 2-iodoacetamide (40 µL, 55 mM) in NH_4HCO_3 (100 mM) at room temperature for 30 min in the dark. This step was followed by washing with H_2O twice and drying again in the speedvac (30 min, 60°C). Trypsin (400 ng) was diluted in NH_4HCO_3 (50 mM) and incubated (30 min, 30 °C). 20 µL of this trypsin solution were given to the gel and incubated (10 min, RT). 40 µL Protease Max (0.01% in 50 mM NH_4HCO_3) were added and incubation was prolonged for 2 h (37 °C). The supernatant was acidified to pH 2 with FA and analyzed by mass spectrometry.

NRPS tripeptide formation assay

The formation of the *D*-Phe-Pro-Leu tripeptide from the interaction of TycA (domain composition A_{Phe}-PCP-E) with one of the TycB1-TycC6 fusion proteins[12] (domain composition C-A_{Pro}-PCP-C-A_{Leu}-PCP-TE) with mutations at the catalytic Ser2168 of the thioesterase (TE) domain was monitored by mass spectrometry. Apo proteins were transformed into the holo form by ppantylation via 4'-phosphopantetheinyl-transferase (PPtase) Sfp and Coenzyme A (CoA). To this end, holo-TycB1-TycC6 (2 µM) and holo-TycA (0.2 µM), as well as the substrate amino acids L-Phe, L-Pro, and L-Leu (each at 1 mM), $MgCl_2$ (20 mM) and ATP (5 mM) were incubated for 3 h at 25 °C. Note that TycA activates L-Phe and racemizes it to D-Phe, which is passed on in the elongation step. The reaction was stopped thermically by incubation at 85 °C for 30 min. The precipitated proteins were separated by centrifugation (4 min, 12000 rpm). The

supernatant containing the peptide was acidified by FA to pH 2 and centrifuged again (3 min, 12000 g). The acidified supernatant was filled into mass vials and the peptide samples were then analyzed by mass spectrometry using an analytic C18 column. Obtained extracted ion chromatograms (EIC) were analyzed in DataAnalysis 4.4 (Bruker Daltonics GmbH, Bremen, GE).

TEV protease cleavage assay

0.2 eq of TEV protease (wildtype or one of the C151 mutants; produced as sfGFP-TEV fusion protein) were incubated with 10 µM of a substrate protein containing a TEV protease cleavage site (amino acid sequence ENLYFQ/G; MBP-ENLYFQG-inteinN) were incubated for the indicated periods of time at 20 °C in PBS buffer at different pHs. The usual pH of 7.4 in PBS was adjusted to pH 8 and 9 by addition of NaOH. The cleavage activity was monitored using SDS-PAGE analysis followed by Coomassie-staining (calculated sizes of the cleavage products MBP-ENLYFQ and G-inteinN were 43.3 kDa and 9.4 kDa, respectively).

Protein *trans*-splicing assay

For protein *trans*-splicing, the N-terminal and C-terminal precursor proteins of the split Gp41-1 intein[13] were added to the reaction in a 3:1 molar ratio (6.6 and 2.2 µM, respectively) and incubated at 37 °C for 1 h in PBS at pH 7.4. The time-dependent formation of splice product was analyzed by removing aliquots for SDS-PAGE analysis and immediately boiling them at 95 °C for 10 min to stop the reaction. The reaction kinetics were calculated following based on densitometric analysis of the Coomassie-stained protein bands using GelAnalyzer (23.1.1). To this end, the obtained values were normalized to the molar masses of proteins. Then the ratio of splice product (SP) to the protein added in deficit (C-terminal precursor P^C) was determined for each time point. The pseudo-first-order reaction was fitted with a simple exponential fit.

Incubation of thymidylate synthase (TS) with 5-F-dUMP

Purified wildtype TS (10 µM) or TS(C146Hcy) (3.3 µM) were incubated with suicide inhibitor 5-F-dUMP (1 mM) for 2 h in PBS buffer with 1 mM TCEP at room temperature. The samples were then acidified to a pH of 1-2 by addition of FA and analyzed by mass spectrometry.

Biochemical assay of TS activity

TS or TS(C146Hcy) (each 3.3 µM) in PBS (pH 7.4) were incubated with combinations of uridine (1 mM), TCEP (1 mM) and 5,10 MTHF (0.25 mM) at 20 °C for 1 h in a final volume of 20 µL. The samples were acidified to pH 2 by addition of formic acid and directly analyzed by LC-MS. For assaying the deprotected TS(C146Hcy), the protected TS(C146Hcy) was first deprotected by TCEP (1 mM; overnight on ice).

In vivo deprotection of sfGFP

Deprotection of the HcyX side chain in live *E. coli* cells was done by addition of DTT (at 0.1 mM to 10 mM) or TCO-NH_2 (at 0.1 mM to 35 mM) to the growth medium 3 h after induction of protein expression. The cultures were incubated for another 3 h at 37 °C. 5 eq. of 4-azidoaniline were added to quench excess DTT or TCO-NH_2 and the cells were subsequently harvested by centrifugation (20 min, 1500 g, 4 °C). The LB medium was discarded thoroughly, and the cells were resuspended in Ni-NTA buffer. Then the lysis of cells and the purification of the His-tagged proteins were performed as described above.

E. coli growth assay using chloramphenicol acetyltransferase (CAT) as selective marker

E. coli BL21 DE3 Gold and *E. coli* T7 SHuffle were transformed with a plasmid (Amp^R) encoding the Cam-cassette, inserted with a fused variant of the Gp41-1 intein harboring either the native S+1 residue or the mutations S+1TAG or S+1A, respectively. The strains intended for

suppression of the amber stop codon were also co-transformed with the plasmid (Kan^R) coding for the orthogonal *Mm* PylRS/tRNA pair. The cells were grown as an overnight culture in LB medium at 37 °C in the presence of ampicillin (100 µg/mL) and kanamycin (50 µg/mL). Overnight cultures were inoculated in LB medium of 10 mL containing antibiotics and induction was performed at OD_{600nm} 0.8 by addition of IPTG (0.4 mM) and L(+)-arabinose (0.2 % w/v). The growth medium of the strains harboring amber-plasmids was further supplemented with HcyX (1 mM). A control sample was grown in the absence of HcyX. Cultures were shaken overnight at 37 °C and were then harvested by centrifugation (4 min, 1500 g, 4 °C). 2 mL of resuspended cells were washed three times with LB medium. Each of the resuspended cells were then adjusted to the same OD and used to inoculate (1:100) a new culture in LB medium supplemented with chloramphenicol (50 µg/mL) additional to Amp and Kan antibiotics, arabinose (0.2 % w/v) and IPTG (0.4 mM), as well as HcyX (1 mM) or no HcyX according to the overnight pre-cultures. These cultures were grown in a volume of 2 mL at 37 °C. Cell growth was monitored over time by measuring the absorption at 600 nm in microtiterplates (Greiner, 96 well, flat, transparent).

E. coli ΔThyA growth assay

E. coli Δ*thyA* strain β1308 (an MG1655 derivative; growth medium supplemented with thymidine (400 µM) of not state otherwise)[14] was transformed with a plasmid (Amp^R) encoding either the *E. coli* thymidylate synthase TS(C146) harboring the native C146 residue or TS(C146TAG) harboring the amber stop codon to incorporate HcyX, respectively. In both cases, a pEVOL plasmid (Kan^R) coding for the orthogonal *Mm* PylRS/tRNA pair was co-transformed. Both strains were grown as separate overnight cultures in LB medium at 37 °C in the presence of ampicillin (100 µg/mL), kanamycin (50 µg/mL) and thymidine (0.4 mM conc.). These pre-cultures were used to inoculate new cultures with the same supplements on the next day. After reaching an OD(600) = 0.8, the culture of the strain encoding TS(C146TAG) was split into two cultures. In all three cultures protein expression was induced by addition of IPTG (0.4 mM) and L(+)-arabinose (0.2 % w/v). At this point, one of the cultures with the strain encoding TS(C146TAG) was further supplemented with HcyX (1 mM) while the other had no HcyX added. The cultures were further incubated overnight at 37 °C. 2 mL of each culture were then used to harvest cells by centrifugation (4 min, 1500 g, 4 °C). Cells were washed four times with LB medium without supplemented thymidine. This step served to remove all excess thymidine from the growth medium. Subsequently, each of the resuspended cell samples was then adjusted to the same OD and used to inoculate (1:100) a new culture in LB medium with the same supplements as in the original overnight pre-culture (i.e., Amp100, Kan50, IPTG (0.4 mM) and arabinose (0.2 % w/v) for all cultures, and additionally HcyX (1 mM) for one of the cultures with the TS(C146TAG) cells). All three cultures (TS(C146), TS(C146TAG)+HcyX, and TS(C146TAG)-HcyX) were then monitored for cell growth at 37 °C by measuring OD(600) in a volume of 0.2 mL in microtiterplates (Greiner, 96 well, flat, transparent).

Disulfide formation and stability assay

sfGFP(N149Hcy) was prepared from sfGFP(N149HcyX) using 4 mM TCO overnight at 37 °C or 2 mM TCEP over 3 h at 20 °C. The deprotecting reagents were removed by purification on a Zeba Spin Desalting Column (7k MWCO; 30-130 µL; Thermo Fisher). To subsequently keep the free thiol of sfGFP(N149Hcy) and sfGFP(N149C) in a reduced form TCEP was added at low concentrations (0.02 mM). Then each of sfGFP(N149Hcy) and sfGFP(N149C), each at 10 µM concentration in PBS buffer (pH 7.4), were incubated with 0.25 mM 2,2'-Dithiobis(5-nitropyridine) (DTNP) for 15 min at room temperature. Then either Cys or Hcy as free amino acids were added to one aliquot of each protein to give a final concentration of 2 mM. The disulfide exchange reactions were allowed to proceed for 30 min at room temperature, at which

point the full conversion of each protein into the disulfide species was confirmed by ESI-MS analysis. To cleave the disulfide by reduction, DTT at excess concentrations (1 mM) was added to each of the reaction mixtures. At indicated time points aliquots were removed and the reaction was stopped by acidification to pH 2 through addition of formic acid, followed by an ESI-MS analysis.

Supporting Table

Table S1: Overview of the recombinant proteins used in this study.

Encoded protein construct	Plasmid	Vector, resistance marker	Amino acid sequence
sfGFP-His	pFM32 (lab collection)	pET21a, Amp	MSKGEELFTGV VPILVELDGD VNGHKFSVRG EGEGDATNGK LTLKFICTTG<br>KLPVPWPTLV TTLTYGVQCF SRYPDHMKRH DFFKSAMPEG YVQERTISFK<br>DDGTYKTRAE VKFEGDTLVN RIELKGIDFK EDGNILGHKL EYNFNSHNVY<br>ITADKQKNGI KANFKIRHNV EDGSVQLADH YQQNTPIGDG PVLLPDNHYL<br>STQSVLSKDP NEKRDHMVLL EFVTAAGITH GGSKGPLEHH HHHH
sfGFP(N149TAG)-His	pPM87 (lab collection)	pET21a, Amp	MSKGEELFTG VVPILVELDG DVNGHKFSVR GEGEGDATNG KLTLKFICTT<br>GKLPVPWPTL VTTLTYGVQC FSRYPDHMKR HDFFKSAMPE GYVQERTISF<br>KDDGTYKTRA EVKFEGDTLV NRIELKGIDF KEDGNILGHK LEYNFNSH(HcyX)V<br>YITADKQKNG IKANFKIRHN VEDGSVQLAD HYQQNTPIGD GPVLLPDNHY<br>LSTQSVLSKD PNEKRDHMVL LEFVTAAGIT HGGSKGPLEH HHHHH
PylRS(P188G, L309A,C348A, Y384F)	pCD35	pEVOL, Cam	MDKKPLNTLI SATGLWMSRT GTIHKIKHHE VSRSKIYIEM ACGDHLVVNN<br>SRSSRTARAL RHHKYRKTCK RCRVSDEDLN KFLTKANEDQ TSVKVKVVSA<br>PTRTKKAMPK SVARAPKPLE NTEAAQAQPS GSKFSPAIPV STQESVSVPA<br>SVSTSISSIS TGATASALVK GNTNPITSMS APVQASAGAL TKSQTDRLEV<br>LLNPKDEISL NSGKPFRELE SELLSRRKKD LQQIYAEERE NYLGKLEREI<br>TRFFVDRGFL EIKSPILIPL EYIERMGIDN DTELSKQIFR VDKNFCLRPM LAPNLYNYAR<br>KLDRALPDPI KIFEIGPCYR KESDGKEHLE EFTMLNFAQM GSGCTRENLE<br>SIITDFLNHL GIDFKIVGDS CMVFGDTLDV MHGDLELSSA VVGPIPLDRE<br>WGIDKPWIGA GFGLERLLKV KHDFKNIKRA ARSESYYNGI STNL
TycB1TycC6-TE(S2168)-His	pJK04 (lab collection, derived from Ref[15])	pTrc99a, Amp	MSVFSKEQVQD MYALTPMQEG MLFHALLDQE HNSHLVQMSI SLQGDLDVGL<br>FTDSLHVLVE RYDVFRTLFL YEKLKQPLQV VLKQRPIPIE FYDLSACDES<br>EKQLRYTQYK RADQERTFHL AKDPLMRVAL FQMSQHDYQV IWSFHHILMD<br>GWCFSIIFDD LLAIYLSLQN KTALSLEPVQ PYSRFINWLE KQNKQAALNY<br>WSDYLEAYEQ KTTLPKKEAA FAKAFQPTQY RFSLNRTLTK QLGTIASQNQ<br>VTLSTVIQTI WGVLLQKYNA AHDVLFGSIV SGRPTDIVGI DKMVGLFINT<br>IPFRVQAKAG QTFSELLQAV HKRTLQSQPY EHVPLYDIQT QSVLKQELID<br>HLLVIENYPL VEALQKKALN QQIGFTITAV EMFEPTNYDL TVMVMPKEEL<br>AFRFDYNAAL FDEQVVQKLA GHLQQIADCV ANNSGVELCQ IPLLTEAETS<br>QLLAKRTETA ADYPAATMHE LFSRQAEKTP EQVAVVFADQ HLTYRELDEK<br>SNQLARFLRK KGIGTGSLVG TLLDRSLDMI VGILGVLKAG GAFVPIDPEL<br>PAERIAYMLT HSRVPLVVTQ NHLRAKVTTP TETIDINTAV IGEESRAPIE SLNQPHDLFY<br>IIYTSGTTGQ PKGVMLEHRN MANLMHFTFD QTNIAFHEKV LQYTTCSFDV<br>CYQEIFSTLL SGGQLYLITN ELRRHVEKLF AFIQEKQISI LSLPVSFLKF IFNEQDYAQS<br>FPRCVKHIIT AGEQLVVTHE LQKYLRQHRV FLHNHYGPSE THVVTTCTMD<br>PGQAIPELPP IGKPISNTGI YILDEGLQLK PEGIVGELYI SGANVGRGYL HQPELTAEKF<br>LDNPYQPGER MYRTGDLARW LPDGQLEFLG RIDHQVKIRG HRIELGEIES<br>RLLNHPAIKE AVVIDRADET GGKFLCAYVV LQKALSDEEM RAYLAQALPE<br>YMIPSFFVTL ERIPVTPNGK TDRRALPKPE GSAKTKADYV APTTELEQKL<br>VAIWEQILGV SPIGIQDHFF TLGGHSLKAI QLISRIQKEC QADVPLRVLF EQPTIQALAA<br>YVEGSAKGNV FSIEPVQKQA YYPVSSAQKR MYILDQFEGV GISYNMPSTM<br>LIEGKLERTR VEAAFQRLIA RHESLRTSFA VVNGEPVQNI HEDVPFALAY<br>SEVTEQEARE LVSSLVQPFD LEVAPLIRVS LLKIGEDRYV LFTDMHHSIS<br>DGVSSGILLA EWVQLYQGDV LPELRIQYKD FAVWQQEFSQ SAAFHKQEAY<br>WLQTFADDIP VLNLPTDFTR PSTQSFAGDQ CTIGAGKALT EGLHQLAQAT<br>GTTLYMVLLT AYNVLLAKYA GQEDIIVGTP ITGRSHADLE PIVGMFVNTL<br>AMRNKPQREK TFSEFLQEVK QNALDAYGHQ DYPFEELVEK LAIARDLSRN<br>PLFDTVFTFQ NSTEEVMTLP ECTLAPFMTD ETGQHAKFDL TFSATEEREE<br>MTIGVEYSTS LFTRETMERF SRHFLTIAAS IVQNPHIRLG EIDMLLPEEK<br>QQILAGFNDT AVSYALDKTL HQLFEEQVDK TPDQAALLFS EQSLTYSELN<br>ERANRLARVL RAKGVGPDRL VAIMAERSPE MVIGILGILK AGGAYVPVDP<br>GYPQERIQYL LEDSNAALLL SQAHLLPLLA QVSSELPECL DLNAELDAGL<br>SGSNLPAVNQ PTDLAYVIYT SGTTGKPKGV MIPHQGIVNC LQWRRDEYGF<br>GPSDKALQVF SFAFDGFVAS LFAPLLGGAT CVLPQEAAAK DPVALKKLMA<br>ATEVTHYYGV PSLFQAILDC STTTDFNQLR CVTLGGEKLP VQLVQKTKEK<br>HPAIEINNEY GPTENSVVTT ISRSIEAGQA ITIGRPLANV QVYIVDEQHH LQPIGVVGEL<br>CIGGAGLARG YLNKPELTAE KFVANPFRPG ERMYKTGDLV KWRTDGTIEY<br>IGRADEQVKV RGYRIEIGEI ESAVLAYQGI DQAVVVARDD DATAGSYLCA<br>YFVAATAVSV SGLRSHLAKE LPAYMIPSYF VELDQLPLSA NGKVDRKALP<br>KPQQSDATTR EYVAPRNATE QQLAAIWQEV LGVEPIGITD QFFELGGHSL<br>KATLLIAKVY EYMQIELPLN LIFQYPTIEK VADFITHKRF ESRYGTAILL NQETARNVFC<br>FTPIGAQSVY YQKLAAEIQG VSLYSFDFIQ DDNRMEQYIA AITAIDPSGP<br>YTLMGY**S**SGG NLAFEVAKEL EERGYGVTDI ILFDSYWKDK AIERTVAETE<br>NDIAQLFAEI GENTEMFNMT QEDFQLYAAN EFVKQSFVRK TVSYVMFHNN<br>LVNTGMTTAA IHLIQSELEA DEEAPVAAKW NESAWANATQ RLLTYSGHGI<br>HSRMLAGDYA SQNASILQNI LQELFILKGS RSHHHHHH
TycB1TycC6-TE(S2168TAG)-His	pCD153	pTrc99a, Amp	same as above, but ncAA encoded by amber stop codon at position of Ser2168
TycB1TycC6-TE(S2168A)-His	pCD155	pTrc99a, Amp	same as above, but with S2168A substitution
SBP-TycA	pJR89 (Ref[15])	pET28a, Kan	MDEKTTGWRG GHVVEGLAGE LEQLRARLEH HPQGQREPMV ANQANLIDNK<br>RELEQHALVP YAQGKSIHQL FEEQAEAFPD RVAIVFENRR LSYQELNRKA

			NQLARALLEK GVQTDSIVGV MMEKSIENVI AILAVLKAGG AYVPIDIEYP RDRIQYILQD SQTKIVLTQK SVSQLVHDVG YSGEVVVLDE EQLDARETAN LHQPSKPTDL AYVIYTSGTT GKPKGTMLEH KGIANLQSFF QNSFGVTEQD RIGLFASMSF DASVWEMFMA LLSGASLYIL SKQTIHDFAA FEHYLSENEL TIITLPPTYL THLTPERITS LRIMITAGSA SSAPLVNKWK DKLRYINAYG PTETSICATI WEAPSNQLSV QSVPIGKPIQ NTHIYIVNED LQLLPTGSEG ELCIGGVGLA RGYWNRPDLT AEKFVDNPFV PGEKMYRTGD LAKWLTDGTI EFLGRIDHQV KIRGHRIELG EIESVLLAHE HITEAVVIAR EDQHAGQYLC AYYISQQEAT PAQLRDYAAQ KLPAYMLPSY FVKLDKMPLT PNDKIDRKAL PEPDLTANQS QAAYHPPRTE TESILVSIWQ NVLGIEKIGI RDNFYSLGGD SIQAIQVVAR LHSYQLKLET KDLLNYPTIE QVALFVKSTT RKSDQGIIAG NVPLTPIQKW FFGKNFTNTG HWNQSSVLYR PEGFDPKVIQ SVMDKIIEHH DALRMVYQHE NGNVVQHNRG LGGQLYDFFS YNLTAQPDVQ QAIEAETQRL HSSMNLQEGP LVKVALFQTL HGDHLFLAIH HLVVDGISWR ILFEDLATGY AQALAGQAIS LPEKTDSFQS WSQWLQEYAN EADLLSEIPY WESLESQAKN VSLPKDYEVT DCKQKSVRNM RIRLHPEETE QLLKHANQAY QTEINDLLLA ALGLAFAEWS KLAQIVIHLE GHGREDIIEQ ANVARTVGWF TSQYPVLLDL KQTAPLSDYI KLTKENMRKI PRKGIGYDIL KHVTLPENRG SLSFRVQPEV TFNYLGQFDA DMRTELFTRS PYSGGNTLGA DGKNNLSPES EVYTALNITG LIEGGELVLT FSYSSEQYRE ESIQQLSQSY QKHLLAIIAH CTEKKEVERT PSDFSVKGLQ MEEMDDIFEL LANTLR
Smt3-Gp41-1Int^{C}(S+1)-Trx-His	pAB64 Ref[13]	pMAL	MKTEEGKLVI WINGDKGYNG LAEVGKKFEK DTGIKVTVEH PDKLEEKFPQ VAATGDGPDI IFWAHDRFGG YAQSGLLAEI TPDKAFQDKL YPFTWDAVRY NGKLIAYPIA VEALSLIYNK DLLPNPPKTW EEIPALDKEL KAKGKSALMF NLQEPYFTWP LIAADGGYAF KYENGKYDIK DVGVDNAGAK AGLTFLVDLI KNKHMNADTD YSIAEAAFNK GETAMTINGP WAWSNIDTSK VNYGVTVLPT FKGQPSKPFV GVLSAGINAA SPNKELAKEF LENYLLTDEG LEAVNKDKPL GAVALKSYEE ELAKDPRIAA TMENAQKGEI MPNIPQMSAF WYAVRTAVIN AASGRQTVDE ALKDAQTNSS SNNNNNNNNN NLGIEGRISE FLVPRGSTRS GYCLDLKTQV QTPQGMKEIS NIQVGDLVLS NTGYNEVLNV FPKSKKKSYK ITLEDGKEII CSEEHLFPTQ TGEMNISGGL KEGMCLYVKE GGHHHHHH
Smt3-Gp41-1Int^{C}(S+1TAG)-Trx-His	pCD52	pBAD, Amp	MGSSMSDSEV NQEAKPEVKP EVKPETHINL KVSDGSSEIF FKIKKTTPLR RLMEAFAKRQ GKEMDSLRFL YDGIRIQADQ TPEDLDMEDN DIIEAHREQI GGMMLKKILK IEELDERELI DIEVSGNHLF YANDILTHN(HcyX)S SDVAGTGSDK IIHLTDDSFD TDVLKADGAI LVDFWAHWCG PCKMIAPILD EIADEYQGKL TVAKLNIDHN PGTAPKYGIR GIPTLLLFKN GEVAATKVGA LSKGQLKEFL DANLAGSEFR SHHHHHH
MBP-Gp41-1Int^{N}-His	pAB74 Ref[16]	pBAD, Amp	MGSSHHHHHH GSGLVPRGSA SMSDSEVNQE AKPEVKPEVK PETHINLKVS DGSSEIFFKI KKTTPLRRLM EAFAKRQGKE MDSLRFLYDG IRIQADQTPE DLDMEDNDII EAHREQIGGM MLKKILKIEE LDERELIDIE VSGNHLFYAN DILTHNSSSD VCGTGSDKII HLTDDSFDTD VLKADGAILV DFWAHWCGPC KMIAPILDEI ADEYQGKLTV AKLNIDHNPG TAPKYGIRGI PTLLLFKNGE VAATKVGALS KGQLKEFLDA NLAGSEFRSH HHHHH
cis-Gp41-1(S+1TAG)-His	pCD65	pMSG, Amp	MKTEEGKLVI WINGDKGYNG LAEVGKKFEK DTGIKVTVEH PDKLEEKFPQ VAATGDGPDI IFWAHDRFGG YAQSGLLAEI TPDKAFQDKL YPFTWDAVRY NGKLIAYPIA VEALSLIYNK DLLPNPPKTW EEIPALDKEL KAKGKSALMF NLQEPYFTWP LIAADGGYAF KYENGKYDIK DVGVDNAGAK AGLTFLVDLI KNKHMNADTD YSIAEAAFNK GETAMTINGP WAWSNIDTSK VNYGVTVLPT FKGQPSKPFV GVLSAGINAA SPNKELAKEF LENYLLTDEG LEAVNKDKPL GAVALKSYEE ELAKDPRIAA TMENAQKGEI MPNIPQMSAF WYAVRTAVIN AASGRQTVDE ALKDAQTNSS SNNNNNNNNN NLGIEGRGTL ETRSGYCLDL KTQVQTPQGM KEISNIQVGD LVLSNTGYNE VLNVFPKSKK KSYKITLEDG KEIICSEEHL FPTQTGEMNI SGGLKEGMCL YVKEGSMLKK ILKIEELDER ELIDIEVSGN HLFYANDILT HN(HcyX)SSDVGTG MSDKIIHLTD DSFDTDVLKA DGAILVDFWA EWCGPCKMIA PILDEIADEY QGKLTVAKLN IDQNPGTAPK YGIRGIPTLL LFKNGEVAAT KVGALSKGQL KEFLDANLAH HHHHH
MG-His$_6$-Gstop-TEVSUMO2(C48A)-(Δ10)SUMO2(C48A, R61C)	pAA36 Ref[4]	pET28a, Kan	MGHHHHHHG(HcyX)E NLYFQGADEK PKEGVKTENN DHINLKVAGQ DGSVVQFKIK RHTPLSKLMK AYAERQGLSM RQIRFRFDGQ PINETDTPAQ LEMEDEDTID VFQQQTGGKT ENNDHINLKV AGQDGSVVQF KIKRHTPLSK LMKAYAERQG LSMRQIRFCF DGQPINETDT PAQLEMEDED TIDVFQQQTG G
MG-His$_6$-TEVSUMO2(C48A, R61stop)-(Δ10)SUMO2(C48A, R61C)	pLK44 Ref[4]	pET28a, Kan	MGHHHHHHENLYFQGADEKPK EGVKTENNDH INLKVAGQDG SVVQFKIKRH TPLSKLMKAY AERQGLSMRQ IRF(HcyX)FDGQPI NETDTPAQLE MEDEDTIDVF QQQTGGKTEN NDHINLKVAG QDGSVVQFKI KRHTPLSKLM KAYAERQGLS MRQIRFCFDG QPINETDTPA QLEMEDEDTI DVFQQQTGG
PylRS(P188G, L309A,C348A, Y384F)	pCD56	pEVOL, Kan	MDKKPLNTLI SATGLWMSRT GTIHKIKHHE VSRSKIYIEM ACGDHLVVNN SRSSRTARAL RHHKYRKTCK RCRVSDEDLN KFLTKANEDQ TSVKVKVVSA PTRTKKAMPK SVARAPKPLE NTEAAQAQPS GSKFSPAIPV STQESVSVPA SVSTSISSIS TGATASALVK GNTNPITSMS APVQASAGAL TKSQTDRLEV LLNPKDEISL NSGKPFRELE SELLSRRKKD LQQIYAEERE NYLGKLEREI TRFFVDRGFL EIKSPILIPL EYIERMGIDN DTELSKQIFR VDKNFCLRPM LAPNLYNYAR KLDRALPDPI KIFEIGPCYR KESDGKEHLE EFTMLNFAQM GSGCTRENLE SIITDFLNHL GIDFKIVGDS CMVFGDTLDV MHGDLELSSA VVGPIPLDRE WGIDKPWIGA GFGLERLLKV KHDFKNIKRA ARSESYYNGI STNL
CAT^{N}-cis-Gp41-1(S+1)-CAT^{C}-His	pCD91	pDrive, Amp	MEKKITGYTT VDISQWHRKE HFEAFQSVAQ CTYNQTVQLD ITAFLKTVKK NKHKFYPAFI HILARLMNAH PEFRMAMKDG ELVIWDSVHP CYTVFHEQTE TFSSLWSEYH DDFRQFLHIY SQDVACYGEN LAYFPKGFIE SGYCLDLKTQ VQTPQGMKEI SNIQVGDLVL SNTGYNEVLN VFPKSKKKSY KITLEDGKEI ICSEEHLFPT QTGEMNISGG LKEGMCLYVK EGSMLKKILK IEELDERELI DIEVSGNHLF YANDILTHNS SSNMFFVSAN PWVSFTSFDL NVANMDNFFA PVFTMGKYYT QGDKVLMPLA IQVHHAVCDG FHVGRMLNEL QQYCDEWQGG AGSHHHHHH

CAT^{N}-cis-Gp41-1(S+1TAG)-CAT^{C}-His	pCD92	pDrive, Amp	MEKKITGYTT VDISQWHRKE HFEAFQSVAQ CTYNQTVQLD ITAFLKTVKK NKHKFYPAFI HILARLMNAH PEFRMAMKDG ELVIWDSVHP CYTVFHEQTE TFSSLWSEYH DDFRQFLHIY SQDVACYGEN LAYFPKGFIE SGYCLDLKTQ VQTPQGMKEI SNIQVGDLVL SNTGYNEVLN VFPKSKKKSY KITLEDGKEI ICSEEHLFPT QTGEMNISGG LKEGMCLYVK EGSMLKKILK IEELDERELI DIEVSGNHLF YANDILTHN(HcyX) SSNMFFVSAN PWVSFTSFDL NVANMDNFFA PVFTMGKYYT QGDKVLMPLA IQVHHAVCDG FHVGRMLNEL QQYCDEWQGG AGSHHHHHH
CAT^{N}-cis-Gp41-1(S+1A)-CAT^{C}-His	pCD94	pDrive, Amp	MEKKITGYTT VDISQWHRKE HFEAFQSVAQ CTYNQTVQLD ITAFLKTVKK NKHKFYPAFI HILARLMNAH PEFRMAMKDG ELVIWDSVHP CYTVFHEQTE TFSSLWSEYH DDFRQFLHIY SQDVACYGEN LAYFPKGFIE SGYCLDLKTQ VQTPQGMKEI SNIQVGDLVL SNTGYNEVLN VFPKSKKKSY KITLEDGKEI ICSEEHLFPT QTGEMNISGG LKEGMCLYVK EGSMLKKILK IEELDERELI DIEVSGNHLF YANDILTHNA SSNMFFVSAN PWVSFTSFDL NVANMDNFFA PVFTMGKYYT QGDKVLMPLA IQVHHAVCDG FHVGRMLNEL QQYCDEWQGG AGSHHHHHH
TS(C146)-His	pCD120	pTrc99a, Amp	MVQYLELMQK VLDEGTQKND RTGTGTLSIF GHQMRFNLQD GFPLVTTKRC HLRSIIHELL WFLQGDTNIA YLHENNVTIW DEWADENGDL GPVYGKQWRA WPTPDGRHID QITTVLNQLK NDPDSRRIIV SAWNVGELDK MALAPCHAFF QFYVADGKLS CQLYQRSCDV FLGLPFNIAS YALLVHMMAQ QCDLEVGDFV WTGGDTHLYS NHMDQTHLQL SREPRPLPKL IIKRKPESIF DYRFEDFEIE GYDPHPGIKA PVAISGSRSH HHHHH
TS(C146TAG)-His	pCD149	pTrc99a, Amp	MVQYLELMQK VLDEGTQKND RTGTGTLSIF GHQMRFNLQD GFPLVTTKRC HLRSIIHELL WFLQGDTNIA YLHENNVTIW DEWADENGDL GPVYGKQWRA WPTPDGRHID QITTVLNQLK NDPDSRRIIV SAWNVGELDK MALAP(HcyX)HAFF QFYVADGKLS CQLYQRSCDV FLGLPFNIAS YALLVHMMAQ QCDLEVGDFV WTGGDTHLYS NHMDQTHLQL SREPRPLPKL IIKRKPESIF DYRFEDFEIE GYDPHPGIKA PVAISGSRSH HHHHH
sfGFP-TEV(C151TAG)-His	pCD151	pET21a, Amp	MASSKGEELF TGVVPILVEL DGDVNGHKFS VRGEGEGDAT NGKLTLKFIC TTGKLPVPWP TLVTTLTYGV QCFSRYPDHM KRHDFFKSAM PEGYVQERTI SFKDDGTYKT RAEVKFEGDT LVNRIELKGI DFKEDGNILG HKLEYNFNSH NVYITADKQK NGIKANFKIR HNVEDGSVQL ADHYQQNTPI GDGPVLLPDN HYLSTQSVLS KDPNEKRDHM VLLEFVTAAG ITHGGSKGPR DYNPISSTIC HLTNESDGHT TSLYGIGFGP FIITNKHLFR RNNGTLLVQS LHGVFKVKNT TTLQQHLIDG RDMIIIRMPK DFPPFPQKLK FREPQREERI CLVTTNFQTK SMSSMVSDTS CTFPSSDGIF WKHWIQTKDG Q(HcyX)GSPLVSTR DGFIVGIHSA SNFTNTNNYF TSVPKNFMEL LTNQEAQQWV SGWRLNADSV LWGGHKVFMV KPEEPFQPVK EATQLMNEGG GLEHHHHHH
sfGFP-TEV(C151A)-His	pCD152	pET21a, Amp	MASSKGEELF TGVVPILVEL DGDVNGHKFS VRGEGEGDAT NGKLTLKFIC TTGKLPVPWP TLVTTLTYGV QCFSRYPDHM KRHDFFKSAM PEGYVQERTI SFKDDGTYKT RAEVKFEGDT LVNRIELKGI DFKEDGNILG HKLEYNFNSH NVYITADKQK NGIKANFKIR HNVEDGSVQL ADHYQQNTPI GDGPVLLPDN HYLSTQSVLS KDPNEKRDHM VLLEFVTAAG ITHGGSKGPR DYNPISSTIC HLTNESDGHT TSLYGIGFGP FIITNKHLFR RNNGTLLVQS LHGVFKVKNT TTLQQHLIDG RDMIIIRMPK DFPPFPQKLK FREPQREERI CLVTTNFQTK SMSSMVSDTS CTFPSSDGIF WKHWIQTKDG QAGSPLVSTR DGFIVGIHSA SNFTNTNNYF TSVPKNFMEL LTNQEAQQWV SGWRLNADSV LWGGHKVFMV KPEEPFQPVK EATQLMNEGG GLEHHHHHH
sfGFP-TEV(C151)-His	pBJ22	pET21a, Amp	MASSKGEELF TGVVPILVEL DGDVNGHKFS VRGEGEGDAT NGKLTLKFIC TTGKLPVPWP TLVTTLTYGV QCFSRYPDHM KRHDFFKSAM PEGYVQERTI SFKDDGTYKT RAEVKFEGDT LVNRIELKGI DFKEDGNILG HKLEYNFNSH NVYITADKQK NGIKANFKIR HNVEDGSVQL ADHYQQNTPI GDGPVLLPDN HYLSTQSVLS KDPNEKRDHM VLLEFVTAAG ITHGGSKGPR DYNPISSTIC HLTNESDGHT TSLYGIGFGP FIITNKHLFR RNNGTLLVQS LHGVFKVKNT TTLQQHLIDG RDMIIIRMPK DFPPFPQKLK FREPQREERI CLVTTNFQTK SMSSMVSDTS CTFPSSDGIF WKHWIQTKDG QCGSPLVSTR DGFIVGIHSA SNFTNTNNYF TSVPKNFMEL LTNQEAQQWV SGWRLNADSV LWGGHKVFMV KPEEPFQPVK EATQLMNEGG GLEHHHHHH
sfGFP(N149C)-His	pCD168	pET21a, Amp	MSKGEELFTGV VPILVELDGD VNGHKFSVRG EGEGDATNGK LTLKFICTTG KLPVPWPTLV TTLTYGVQCF SRYPDHMKRH DFFKSAMPEG YVQERTISFK DDGTYKTRAE VKFEGDTLVN RIELKGIDFK EDGNILGHKL EYNFNSHCVY ITADKQKNGI KANFKIRHNV EDGSVQLADH YQQNTPIGDG PVLLPDNHYL STQSVLSKDP NEKRDHMVLL EFVTAAGITH GGSKGPLEHH HHHH

Supporting References

[1] C. C. Cho, L. R. Blankenship, X. Ma, S. Xu, W. Liu, *J Mol Biol*, **2022**, *434*, 167453.

[2] K. Tori, B. Dassa, M. A. Johnson, M. W. Southworth, L. E. Brace, Y. Ishino, S. Pietrokovski, F. B. Perler, *J Biol Chem*, **2010**, *285*, 2515-2526.

[3] K. F. Geoghegan, H. B. Dixon, P. J. Rosner, L. R. Hoth, A. J. Lanzetti, K. A. Borzilleri, E. S. Marr, L. H. Pezzullo, L. B. Martin, P. K. LeMotte, A. S. McColl, A. V. Kamath, J. G. Stroh, *Anal Biochem*, **1999**, *267*, 169-184.

[4] M. Reille-Seroussi, P. Meyer-Ahrens, A. Aust, A. L. Feldberg, H. D. Mootz, *Angew Chem Int Ed Engl*, **2021**, *60*, 15972-15979.

[5] K. Gorska, A. Manicardi, S. Barluenga, N. Winssinger, *Chem Commun (Camb)*, **2011**, *47*, 4364-4366.

[6] Y. M. Choi, *WIPO*, **2014**, 142519.

[7] R. Uprety, J. Luo, J. Liu, Y. Naro, S. Samanta, A. Deiters, *Chembiochem*, **2014**, *15*, 1793-1799.

[8] C. Lherbet, J. W. Keillor, *Org Biomol Chem*, **2004**, *2*, 238-245.

[9] A. Tuley, Y. J. Lee, B. Wu, Z. U. Wang, W. R. Liu, *Chem Commun (Camb)*, **2014**, *50*, 7424-7426.

[10] Y. Ge, X. Fan, P. R. Chen, *Chem Sci*, **2016**, *7*, 7055-7060.

[11] J. S. Wesalo, J. Luo, K. Morihiro, J. Liu, A. Deiters, *Chembiochem*, **2020**, *21*, 141-148.

[12] H. D. Mootz, D. Schwarzer, M. A. Marahiel, *Proc Natl Acad Sci U S A*, **2000**, *97*, 5848-5853.

[13] J. K. Böcker, W. Dörner, H. D. Mootz, *Chem Commun (Camb)*, **2019**, *55*, 1287-1290.

[14] V. Döring, P. Marlière, *Genetics*, **1998**, *150*, 543-551.

[15] J. Rüschenbaum, W. Steinchen, F. Mayerthaler, A. L. Feldberg, H. D. Mootz, *Angew Chem Int Ed Engl*, **2022**, *61*, e202212994.

[16] A. L. Bachmann, H. D. Mootz, *J Biol Chem*, **2015**, *290*, 28792-28804.
